# Nirmatrelvir-Ritonavir for Acute COVID-19 in Patients With Cardiovascular Disease and Postacute Sequelae of SARS-CoV-2 Infection

**DOI:** 10.1016/j.jacadv.2024.100961

**Published:** 2024-06-26

**Authors:** Rushin Patel, Sourbha S. Dani, Sumanth Khadke, Ashish Kumar, Javaria Ahmad, Anu Mariam Saji, Jui Shah, Neev Mehta, Kenneth Wener, Daniel P. McQuillen, George Abraham, Jeremy Faust, Jason Maley, Smita Patel, Janet Mullington, Robert M. Wachter, Anne Mosenthal, Paul E. Sax, Sarju Ganatra

**Affiliations:** a Department of Internal Medicine, Lahey Hospital and Medical Center, Beth Israel Lahey Health, Burlington, Massachusetts, USA; b Department of Cardiovascular Medicine, Lahey Hospital and Medical Center, Beth Israel Lahey Health, Burlington, Massachusetts, USA; c Department of Internal Medicine, Cleveland Clinic Akron General, Akron, Ohio, USA; d Department of Internal Medicine, Saint Vincent Hospital, Worcester, Massachusetts, USA; e Department of Infectious Diseases, Lahey Hospital and Medical Center, Beth Israel Lahey Health, Burlington, Massachusetts, USA; f Division of Infectious Disease, Department of Medicine, Saint Vincent Hospital, Worcester, Massachusetts, USA; g Department of Emergency Medicine, Brigham and Women's Hospital, Boston, Massachusetts, USA; h Department of Neurology, Beth Israel Deaconess Medical Center, Boston, Massachusetts, USA; i Department of Psychiatry, Lahey Hospital and Medical Centre, Beth Israel Lahey Health, Burlington, Massachusetts, USA; j Department of Medicine, University of California-San Francisco, San Francisco, California, USA; k Department of Academic Affairs, Lahey Hospital and Medical Center, Tufts University School of Medicine, Burlington, Massachusetts, USA; l Division of Infectious Disease, Department of Medicine, Brigham and Women's Hospital and Harvard Medical School, Boston, Massachusetts, USA

**Keywords:** cardiovascular disease, health care utilization, nirmatrelvir-ritonavir, postacute sequelae of SARS-CoV-2 infection, long COVID

## Abstract

**Background:**

There is limited evidence of association of nirmatrelvir-ritonavir (NMV-r) and incidence of postacute sequelae of SARS-CoV-2 infection (PASC) in patients with pre-existing cardiovascular disease (CVD).

**Objectives:**

The objective of this study was to assess the association of NMV-r in nonhospitalized, vaccinated patients with pre-existing CVD and occurrence of PASC.

**Methods:**

We conducted a retrospective cohort study utilizing the TriNetX research network, including vaccinated patients with pre-existing CVD who developed COVID-19 between December 2021 and December 2022. Two cohorts were created based on NMV-r administration within 5 days of diagnosis: NMV-r and non-NMV-r cohort. The main outcome was presence of PASC, assessed between 30 to 90 days and 90 to 180 days after index COVID-19 infection. After propensity score matching, both cohorts were compared using t-test and chi-square test for continuous and categorical variables, respectively.

**Results:**

A total of 26,953 patients remained in each cohort after propensity score matching. Broadly defined PASC occurred in 6,925 patients (26%) in the NMV-r cohort vs 8,150 patients (30.6%) in the non-NMV-r cohort (OR: 0.80; 95% CI: 0.76-0.82; *P* < 0.001) from 30 to 90 days and in 6,692 patients (25.1%) as compared to 8,910 patients (33.5%) (OR: 0.25, 95% CI: 0.23-0.29; *P* < 0.001) from 90 to 180 days. Similarly, narrowly defined PASC occurred in 5,335 patients (20%) in the NMV-r cohort vs 6,271 patients (23.6%) in the non-NMV-r cohort between 30 and 90 days (OR: 0.81, 95% CI: 0.78-0.84, *P* < 0.001) and in 5,121 patients (19.2%) as compared to 6,964 patients (26.1%) (OR: 0.67, 95% CI: 0.64-0.70, *P* < 0.001) between 90 and 180 days.

**Conclusions:**

NMV-r in nonhospitalized vaccinated patients with pre-existing CVD with COVID-19 was associated with a reduction in PASC and health care utilization.

The clinical presentations from COVID-19 caused by the SARS-CoV-2 virus can range from an asymptomatic or mildly symptomatic disease managed in the outpatient setting to severe illness requiring hospitalization. In addition to acute illness, COVID-19 can result in a lingering long-term cluster of symptoms lasting for months.^1^ Numerous terms, including “long COVID,” “post-COVID condition,” and postacute sequelae of SARS-CoV2 infection (PASC), have been used to describe these syndromes.^2^ As of July 2023, 659 million people worldwide have recovered from COVID-19.^3^ The reported prevalence of PASC is highly varied due to heterogeneity in the definition and different populations studied; however, even with the lower prevalence estimate, the numbers suggest an overwhelming global disease burden, making it a condition with enormous medical, public health, and economic consequences.

Patients with cardiovascular disease (CVD) have a higher risk of adverse outcomes in the acute phase of COVID-19.^4^ It is being recognized that CVD and some of its risk factors, including older age, higher body mass index, smoking, diabetes, and ischemic heart disease, are also associated with an increased risk of developing PASC.^5^ Additionally, the commonly reported symptoms of PASC include chest pain, shortness of breath, palpitations, and fatigue.^6^ Such overlapping symptoms are likely to be of higher severity in patients with pre-existing CVD. It also further complicates the diagnosis process and likely either delays the diagnosis or may even get misdiagnosed given the difficulty in differentiating the etiology of the symptoms, whether they represent underlying CVD or related to PASC or, in many scenarios, both. In addition to the strain on an individual patient, in patients with pre-existing CVD, such symptoms may lead to more diagnostic tests and increased healthcare resource utilization, which may burden scarce health care resources.

Advances in vaccination, therapies, and the impact of widespread immunity acquired from prior infection have markedly lowered the case-fatality rate of COVID-19. Additionally, the availability of effective outpatient therapeutics, including nirmatrelvir plus ritonavir (NMV-r),^7^ molnupiravir,^8^ remdesivir,^9^ and various monoclonal antibodies, are significant advances.^10^ While the efficacy of these treatments in lowering the risk of acute COVID-19 has been well-established, the published data in peer-reviewed literature regarding their effectiveness in reducing the frequency of PASC are limited, barring a recent Department of Veterans Affairs study meta-analysis showing an association of early treatment of COVID-19 and decreased risk of PASC.^11^

NMV-r is a favored treatment for outpatient management of COVID-19 based on the results of a pivotal placebo-controlled trial in high-risk unvaccinated patients with mild-moderate disease.^7^ In this study, NMV-r resulted in an 89% reduction in risk of hospitalization or all-cause mortality compared to the placebo, leading to the emergency use authorization of the drug. Subsequently, in several observational studies, NMV-r treatment was associated with reduced all-cause mortality, emergency room visits, and hospitalizations in broader patient groups, including relatively young (18-50 years of age) high-risk vaccinated patients with COVID-19 such as those with CVD.^12^

Given the frequency and consequences of PASC in patients with high-risk factors, it is crucial to determine whether antiviral therapy is effective in preventing PASC. Accumulating evidence suggests that viral particles persist throughout the human body after the resolution of the acute infection.^13^^,^^14^ This has been thought to be one of the potential mechanisms behind the pathogenesis of PASC.^15^ Intuitively, treatment with NMV-r could potentially reduce the incidence of PASC by facilitating viral clearance. While ideally, these data would come from prospective randomized placebo-controlled clinical trials; such studies have not yet reported these outcomes with sufficient follow-up to answer this question. To address this data gap, we utilized the electronic health records (EHR)-based, curated real-world data of the TriNetX research network to describe the frequency of PASC-associated symptoms among those treated vs not treated with NMV-r.

## Methods

### Study oversight

All the authors were involved in data analysis, writing, and reviewing the manuscript. Institutional review board approval was exempted from Lahey Clinic Institutional Review Board as deidentified data were used for analysis. The study findings are reported per the Strengthening the Reporting of Observational Studies in Epidemiology guidelines for cohort studies.

### Data source and study setting

This study utilized the TriNetX Analytics Network-Research Network, a multicenter federated health research network using deidentified data from EHRs from participating health care organizations, including academic medical centers, specialty physician practices, and community hospitals. The research network contains data on more than 110 million patients. While the data are in aggregate deidentified form, the built-in analytics allows for the generation of patient-level data for cohort selection and matching, analyzing incidence and prevalence of events in a cohort, and comparing characteristics and outcomes between matched cohorts. More information on the database can be found online.^16^

### Study population and design

The TriNetX research network was searched, and data curation was performed on July 1, 2023. A comparative retrospective cohort study was conducted, which included nonhospitalized patients ≥18 years of age with pre-existing CVD (defined as the presence of 1 or more comorbidities including hypertension, hyperlipidemia, type 2 diabetes mellitus, ischemic heart disease, cerebrovascular disease, cardiomyopathy/heart failure, arrhythmia) who were vaccinated and subsequently developed COVID-19. Patients were selected irrespective of whether it was an initial or subsequent breakthrough COVID-19 infection between December 1, 2021, and December 31, 2022, at least 4 weeks after vaccination, albeit completion of initial series or administration of booster dose. Key exclusion criteria were treatment with a monoclonal antibody, convalescent plasma, or molnupiravir for the index case of COVID-19 and hospitalization for COVID-19. Patients were further categorized based on whether they were prescribed NMV-r within 5 days of diagnosis.

Patients with PASC were identified using 2 definitions based on the International Classification of Diseases-10th Revision code. Validated diagnostic, procedure, and laboratory codes were utilized to define the vaccination status and COVID-19 diagnosis. Identification of patients who were prescribed NMV-r was completed using the National Library of Medicine RxNorm terminology.

Cohorts were matched using propensity score matching using multiple baseline characteristics as deemed clinically significant. Outcomes were analyzed from 30 to 90 days and between 90 and 180 days after the index diagnosis of COVID-19.

### Study endpoints/variables

#### Main composite endpoint

Since no validated diagnostic tests exist, PASC is defined by patient-reported symptoms. To account for the lack of universal applicability of a standard definition and the wide range of estimates on the prevalence of PASC after COVID-19, we defined PASC both broadly and narrowly, using prior published reports.^17^, ^18^, ^19^

1) Broadly defined PASC: This included the presence of 1 or more symptoms, including a list of the constitutional, cardiorespiratory, gastrointestinal, musculoskeletal, and nervous system, and/or mood/cognitive disorder symptoms.^17^^,^^18^
2) Narrowly defined PASC: This included: 1) persistent fatigue with myalgia or mood disorder symptoms; and/or 2) cognitive disorder; and/or 3) respiratory symptoms.^19^

Other outcomes, including complications and health care utilization, were also defined using validated codes.

In addition to the absence of a specific diagnostic test, there is a lack of agreement regarding how long symptoms persist after COVID-19 qualify as PASC. Therefore, the outcomes were analyzed beyond the first 30 days, beyond the first 90 days, and up to 180 days following the index diagnosis of COVID-19.

Both broadly defined PASC as well as narrowly defined PASC included new-onset symptoms rather than pre-existing similar symptoms.

#### Other endpoints

Secondary endpoints included individual symptoms of PASC. We also assessed health care utilization, including diagnostic imaging and cardiovascular testing.

### Statistical analysis

Nonhospitalized, vaccinated patients who developed COVID-19 at least 4 weeks after vaccination were divided into 2 cohorts based on their use of NMV-r within 5 days of diagnosis. These 2 cohorts (NMV-r and non-NMV-r cohorts) were compared using independent sample t-tests for continuous variables, reported as mean (range). Categorical variables are reported as counts (%) and compared using the chi-square test. To control for baseline differences in the patient cohorts, we performed 1:1 propensity score matching for characteristics of clinical relevance utilizing a built-in algorithm that uses the greedy nearest-neighbor algorithm with a caliper of 0.1 pooled standard mean difference. First, symptoms of PASC seen between 30 and 90 days after developing COVID-19 were assessed. Subsequently, symptoms of PASC seen between 90 and 180 days were also assessed. Any characteristic with a standardized mean difference between cohorts lower than 0.1 was considered well-matched. After propensity matching, ORs with 95% CIs were calculated for primary and secondary outcomes using the chi-square test for the measures of association. Relative risk reduction was calculated as the division of the absolute risk reduction between the treatment (NMV-r) and control (non-NMV-r) cohorts by the absolute risk of the control group. E-values were also calculated for OR.^20^^,^^21^ Statistical analyses were completed using the TriNetX online platform using R for statistical computing.

Further information can be obtained in the Supplemental Appendix.

## Results

### Study population

**Figure 1:**
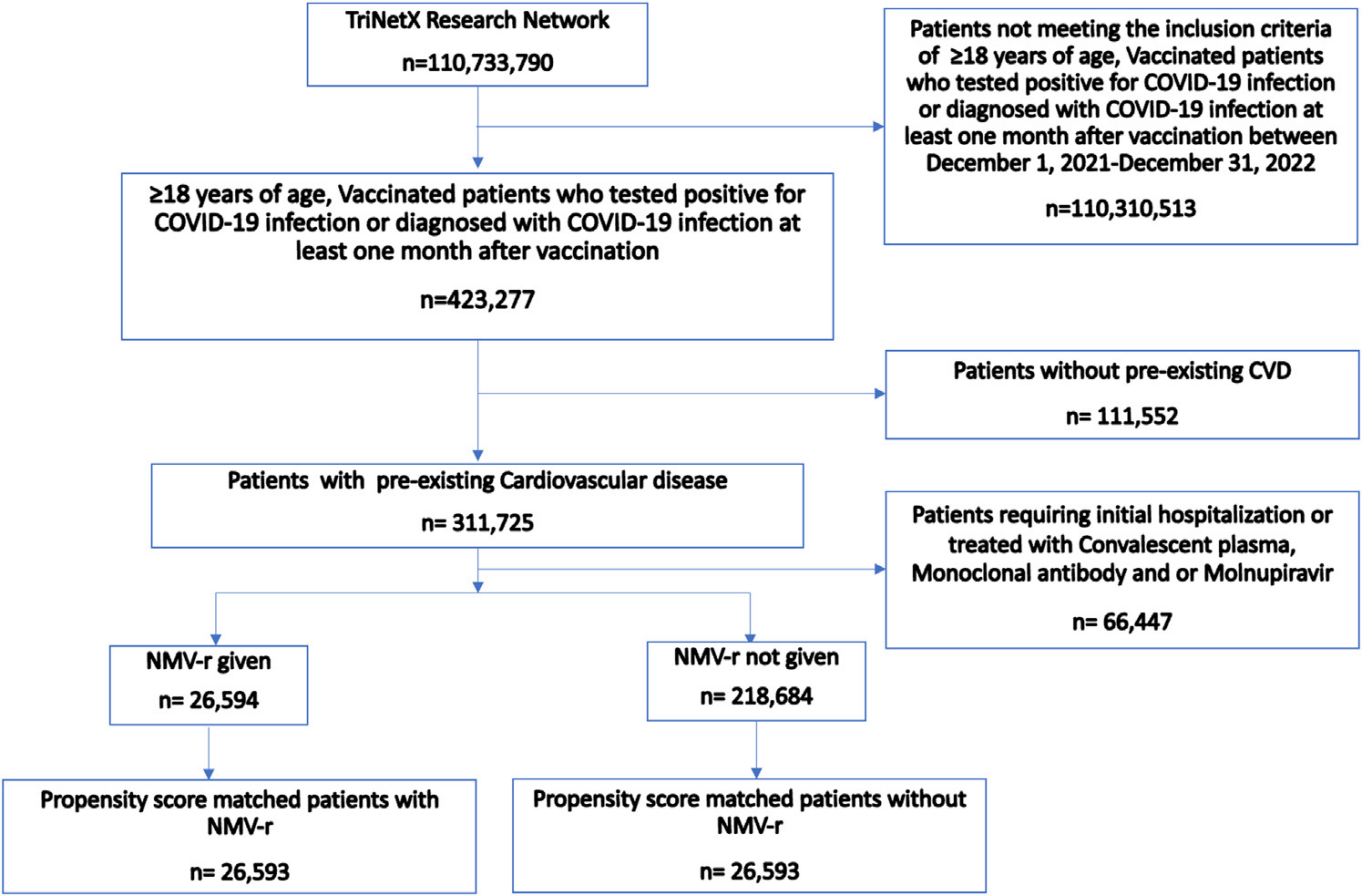
**CONSORT Diagram** This figure illustrates the proportion of vaccinated, nonhospitalized patients who tested positive for SARS-CoV-2 infection or were diagnosed with COVID-19 stratified by the use of NMV-r. CONSORT = Consolidated Standards of Reporting Trials; HCO = health care organization; NMV-r = nirmatrelvir plus ritonavir.

A total of 245,278 vaccinated individuals who tested positive for COVID-19 and were not hospitalized during the study were identified. Of these, 26,594 were treated with NMV-r within 5 days of diagnosis and had sufficient follow-up for inclusion, and 218,684 were not treated with NVM-r. After propensity score matching, 26,593 patients were included in each cohort (Figure 1).

### Patient demographics

Table 1 outlines the baseline characteristics of each cohort before and after propensity matching. Before propensity matching, the mean age of patients treated with NMV-r was higher (mean age 62.4 ± 14.5 years vs 57.6 ± 16.8 years; SD: 0.30), and there were racial differences—the proportion of the White adults was higher in the NMV-r cohort compared to non-NMV-r cohort (79.2% in NMV-r vs 72.4%). In contrast, the proportion of Black adults was higher in the non-NMV-r cohort (14.9% in non-NMV-r vs 10.2% in NMV-r). Majority of the patients in the NMV-r and non-NMV-r cohorts were women (61.2% vs 61.1%). In addition, patients in the NMV-r cohort had a higher prevalence of hypertension and hyperlipidemia. In comparison, patients in the non-NMV-r cohort had a higher prevalence of heart failure and atrial fibrillation/flutter. The proportion of metformin use was similar in both cohorts. In the NMV-r cohort, the prevalence was also higher for neoplasms, systemic connective tissue disorders, and chronic lower respiratory disease. All baseline characteristics between the 2 cohorts, including health care utilization before the index event, were propensity matched, with no residual difference (standard mean difference for all included covariates was <0.1). This has been shown in Table 1.

**Table 1:** Baseline Characteristics

	Before Propensity Matching			After Propensity Matching		
	NMV-r Cohort (n = 26,594)	Non-NMV-r Cohort (n = 218,684)	SMD	NMV-r Cohort (n = 26,593)	Non-NMV-r Cohort (n = 26,593)	SMD
Demographics						
Age (y)	62.4 ± 14.5	57.6 ± 16.8	0.30	62.4 ± 14.5	62.4 ± 14.9	0.00
Women	16,265 (61.2%)	133,601 (61.1%)	0.00	16,264 (61.2%)	16,194 (60.9%)	0.00
Caucasian	21,071 (79.2%)	158,398 (72.4%)	0.15	21,070 (79.2%)	21,219 (79.8%)	0.01
African American	2,707 (10.2%)	32,588 (14.9%)	0.14	2,707 (10.2%)	2,646 (9.9%)	0.00
Non-Hispanic/Latino	22,066 (83.0%)	165,896 (75.9%)	0.17	22,065 (83.0%)	22,034 (82.9%)	0.00
Prior health care utilization						
Ambulatory visit	25,662 (96.5%)	188,011 (86.0%)	0.37	25,661 (96.5%)	25,572 (96.2%)	0.01
ER visit	12,874 (48.4%)	115,113 (52.6%)	0.08	12,874 (48.4%)	12,770 (48.0%)	0.00
Hospital inpatient services	4,453 (16.7%)	48,325 (22.1%)	0.13	4,453 (16.7%)	4,381 (16.5%)	0.00
Hospital observation services	2,618 (9.8%)	26,230 (12%)	0.06	2,618 (9.8%)	2,521 (9.5%)	0.01
Critical care services	1,082 (4.1%)	13,917 (6.4%)	0.10	1,082 (4.1%)	1,065 (4.0%)	0.00
Comorbidities						
Hypertension	17,930 (67.4%)	132,857 (60.8%)	0.13	17,929 (67.4%)	17,814 (67.0%)	0.00
Hyperlipidemia	18,496 (69.5%)	122,283 (55.9%)	0.28	18,495 (69.5%)	18,483 (69.5%)	0.00
Diabetes mellitus	7,261 (27.3%)	57,693 (26.3%)	0.02	7,261 (27.3%)	7,067 (26.6%)	0.01
Chronic lower respiratory disease	8,226 (30.9%)	62,538 (28.6%)	0.05	8,226 (30.9%)	8,160 (30.7%)	0.00
Chronic kidney disease	2,653 (10%)	29,147 (13.3%)	0.10	2,653 (10%)	2,591 (9.7%)	0.00
Atrial fibrillation/flutter	1,765 (6.6%)	21,297 (9.7%)	0.11	1,765 (6.6%)	1,734 (6.5%)	0.00
Ischemic heart disease	5,098 (19.2%)	42,613 (19.5%)	0.00	5,098 (19.2%)	5,127 (19.3%)	0.00
Heart failure	1,690 (6.4%)	21,456 (9.8%)	0.12	1,690 (6.4%)	1,691 (6.4%)	<0.001
Ischemic stroke	898 (3.4%)	9,486 (4.3%)	0.05	898 (3.4%)	838 (3.15%)	0.01
Malignancy	9,462 (35.6%)	80,559 (36.8%)	0.02	9,462 (35.6%)	9,349 (35.2%)	0.00
Demyelinating disease	281 (1.1%)	2,197 (1.0%)	0.00	281 (1.1%)	280 (1.1%)	<0.001
Systematic connective tissue disorder	1,181 (4.4%)	9,862 (4.5%)	0.00	1,181 (4.4%)	1,192 (4.5%)	0.00
BMI >30 kg/m^2^	7,427 (27.9%)	65,044 (29.7%)	0.04	7,427 (27.9%)	7,353 (27.7%)	0.00
Medications						
Beta-blockers	9,677 (36.4%)	82,221 (37.6%)	0.02	9,677 (36.4%)	9,503 (35.7%)	0.01
Diuretics	9,182 (34.5%)	76,884 (35.2%)	0.01	9,182 (34.5%)	8,978 (33.8%)	0.01
ACE inhibitors	6,488 (24.4%)	50,539 (23.1%)	0.03	6,487 (24.4%)	6,467 (24.3%)	0.00
Angiotensin receptor blocker	6,010 (22.6%)	41,480 (19%)	0.09	6,009 (22.6%)	5,880 (22.1%)	0.01
Aspirin	6,350 (23.9%)	58,064 (26.6%)	0.06	6,350 (23.9%)	6,228 (23.4%)	0.01
Anticoagulants	7,114 (26.8%)	72,284 (33.1%)	0.13	7,114 (26.8%)	7,061 (26.6%)	0.00
Statin	13,877 (52.2%)	95,314 (43.6%)	0.17	13,876 (52.2%)	13,851 (52.1%)	0.00
Immune suppressants	1,045 (3.9%)	13,076 (6%)	0.09	1,045 (3.9%)	1,409 (5.3%)	0.06
Antineoplastics	2,879 (10.8%)	25,818 (11.8%)	0.03	2,879 (10.8%)	2,885 (10.8%)	0.00
Antidepressant	10,530 (39.6%)	86,424 (39.5%)	0.00	10,529 (39.6%)	10,504 (39.5%)	0.00
Metformin	4,709 (17.7%)	34,377 (15.7%)	0.05	4,709 (17.7%)	4,565 (17.2%)	0.01
Laboratory tests						
Hemoglobin (g/dL)	13.7 ± 1.7 (n = 22,527)	13.4 ± 1.9 (n = 177,310)	0.20	13.7 ± 1.7 (n = 22,526)	13.6 ± 1.7 (n = 22,983)	0.07
Platelets (10 × 3/μL)	252.5 ± 73.4 (n = 23,299)	251.5 ± 77.6 (n = 179,524)	0.01	252.5 ± 73.4 (n = 23,298)	250.0 ± 74.0 (n = 23,265)	0.03
Creatinine (mg/dL)	0.9 ± 0.3 (n = 24,335)	1.0 ± 2.0 (n = 184,812)	0.08	0.9 ± 0.3 (n = 24,334)	1.0 ± 1.7 (n = 24,263)	0.05
Total cholesterol (mg/dL)	177.8 ± 41.9 (n = 21,577)	176.5 ± 44.4 (n = 149,764)	0.03	177.8 ± 41.9 (n = 21,576)	178.5 ± 44.4 (n = 21,712)	0.01
LDL (mg/dL)	100.5 ± 35.6 (n = 22,075)	100.0 ± 36.4 (n = 152,028)	0.01	100.5 ± 35.6 (n = 22,074)	100.8 ± 36.8 (n = 22,130)	0.00
Triglyceride (mg/dL)	132.6 ± 82.9 (n = 22,134)	135.1 ± 100.5 (n = 152,773)	0.02	132.6 ± 82.9 (n = 22,133)	135.3 ± 87.4 (n = 22,161)	0.03
Hemoglobin A1C (%)	6.1 ± 1.4 (n = 17,788)	6.2 ± 1.5 (n = 120,501)	0.03	6.1 ± 1.4 (n = 17,787)	6.2 ± 1.5 (n = 17,688)	0.01
Left ventricular ejection fraction (%)	61.2 ± 9.1 (n = 2,333)	59.5 ± 10.1 (n = 18,989)	0.17	61.2 ± 9.1 (n = 2,333)	60.6 ± 9.4 (n = 2,294)	0.05

Values are mean ± SD or n (%) unless otherwise indicated.

ACE = angiotensin-converting enzyme; BMI = body mass index; LDL = low-density lipoprotein; SMD = standard mean difference.

### Main outcome

#### Between 30 and 90 days postinfection

For broadly defined PASC, vaccinated patients treated with NMV-r within 5 days of developing COVID-19 had lower odds and reduced risk of developing persistent or new PASC. For example, broadly defined PASC occurred in 6,925 (26%) patients in the NMV-r cohort, as compared to 8,150 (30.6%) patients in the non-NMV-r cohort (OR: 0.80; 95% CI: 0.77-0.83; *P* < 0.001) between 30 and 90 days. Similarly, for a narrow definition of PASC to 3 symptom clusters, the use of NMV-r within 5 days of development of COVID-19 was associated with lower odds and reduced risk of development of PASC, which was recorded in 5,335 (20%) patients in the NMV-r cohort and 6,271 (23.6%) of patients in the non-NMV-r cohort between 30 and 90 days (OR: 0.81, 95% CI: 0.78-0.85, *P* < 0.001).

#### Between 90 and 180 days postinfection

Even upon modifying the timeline for developing new or persistent PASC between 90 and 180 days, patients treated with NMV-r within 5 days of diagnosis had lower odds and reduced risk of having persistent or new PASC. Broadly defined PASC occurred in 6,692 patients (25.1%) in the NMV-r cohort, as compared to 8,910 patients (33.5%) in the non-NMV-r cohort (OR: 0.67; 95% CI: 0.64-0.69; *P* < 0.001) from 90 to 180 days. In addition, narrowly defined PASC based on 3 symptom cluster was reported in 5,121 (19.2%) in the NMV-r cohort as compared to 6,964 (26.1%) patients in the non-NMV-r cohort (OR: 0.67, 95% CI: 0.64-0.70, *P* < 0.001).

### Other outcomes

#### Between 30 and 90 days postinfection

**Figure 2:**
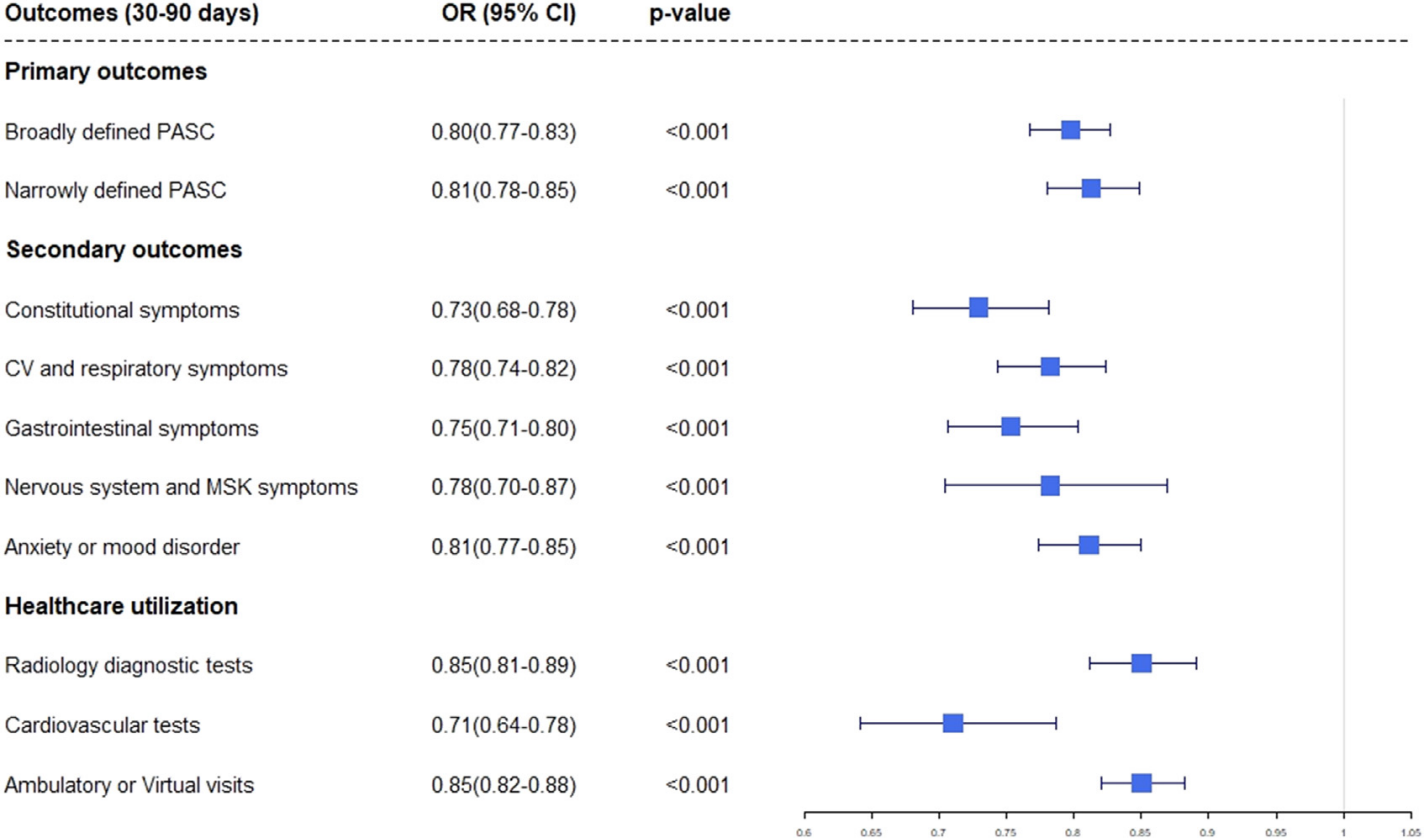
**Forest Plot of Postacute Sequelae of SARS-CoV-2 Infection Outcomes at 30 to 90 Days After the Index COVID-19 Infection** This Forest plot depicts the primary and secondary outcomes within 30 to 90 days after the diagnosis of COVID-19. CV = cardiovascular; MSK = musculoskeletal; PASC = postacute sequelae of SARS-CoV-2 infection.

Patients who had been treated with NMV-r within 5 days of infection with COVID-19 had lower odds and reduced risk of having cardiovascular and respiratory symptoms (OR: 0.78, 95% CI: 0.74-0.82, *P* < 0.001); gastrointestinal symptoms (OR: 0.75, 95% CI: 0.70-0.80, *P* < 0.001); and lower odds and reduced risk of anxiety/mood disorders (OR: 0.811, 95% CI: 0.77-0.85, *P* < 0.001), nervous system (OR: 0.78, 95% CI: 0.70-0.87, *P* < 0.001), and constitutional symptoms (OR: 0.73, 95% CI: 0.68-0.78, *P* < 0.001), between 30 and 90 days postinfection. Cardiovascular testing was performed less frequently in the NMV-r cohort. The patients in the NMV-r cohort had lower odds and risk of health care utilization in the form of radiological tests (OR: 0.85, CI: 0.81-0.89, *P* < 0.001); cardiovascular tests (OR: 0.71, 95% CI: 0.64-0.78, *P* < 0.001); and ambulatory or virtual visits (OR: 0.85, 95% CI: 0.82-0.88, *P* < 0.001). This has been depicted in Table 2 and Figure 2.

**Table 2:** Postacute Sequelae of SARS-CoV-2 Infection Outcomes at 30 to 90 Days After the Index COVID-19 Infection

						E Values		
Outcomes (30-90 d)	NMV-r Cohort (n = 26,593)	Non-NMV-r Cohort (n = 26,593)^a^	Risk Difference (95% CI)	Relative Risk Reduction (95% CI)	OR (95% CI)	OR	Lower CI for OR	*P* Value
Primary outcome								
Broadly defined PASC	6,925 (26%)	8,150 (30.6%)	−0.046 (−0.054 to −0.038)	15% (13%-17%)	0.797 (0.767–0.827)	1.49	1.54	<0.001
Narrowly defined PASC	5,335 (20%)	6,271 (23.5%)	−0.035 (−0.042 to −0.028)	15% (12%-17%)	0.813 (0.780–0.848)	1.46	1.39	<0.001
Secondary outcomes								
Individual symptoms								
Constitutional symptoms	1,499 (5.6%)	2,014 (7.5%)	−0.019 (−0.024 to −0.015)	25% (20%-30%)	0.729 (0.680–0.781)	2.09	2.3	<0.001
CV and respiratory symptoms	3,015 (11.3%)	3,738 (14%)	−0.027 (−0.033 to −0.022)	19% (15%-23%)	0.782 (0.743–0.823)	1.88	2.03	<0.001
Gastrointestinal symptoms	1,751 (6.5%)	2,276 (8.5%)	−0.020 (−0.024 to −0.015)	23% (18%-27%)	0.753 (0.706–0.803)	1.99	2.18	<0.001
Nervous system and MSK symptoms	629 (2.3%)	799 (3%)	−0.006 (−0.009 to −0.004)	21% (13%-29%)	0.782 (0.704–0.869)	1.88	2.19	<0.001
Anxiety/mood disorder	3,650 (13.7%)	4,362 (16.4%)	−0.027 (−0.033 to −0.021)	16% (13%-19%)	0.811 (0.773–0.850)	1.77	1.91	<0.001
Health care utilization								
Radiology diagnostic tests	3,782 (14.2%)	4,341 (16.3%)	−0.021 (−0.027 to −0.015)	13% (9%-16%)	0.850 (0.811–0.891)	1.63	1.77	<0.001
CV tests (echocardiogram and ambulatory rhythm monitors)	650 (2.4%)	906 (3.4%)	−0.010 (−0.012 to −0.007)	28% (20%-35%)	0.710 (0.641–0.787)	2.17	2.49	<0.001
AMB/Virtual visit	17,581 (66.1%)	18,521 (69.6%)	−0.035 (−0.043 to −0.027)	5% (4%-6%)	0.850 (0.820–0.882)	1.39	1.44	<0.001

Values are n (%) unless otherwise indicated.

AMB = ambulatory; CV = cardiovascular; ER = emergency room; MSK = musculoskeletal.

a After propensity score matching.

#### Between 90 and 180 days postinfection

**Figure 3:**
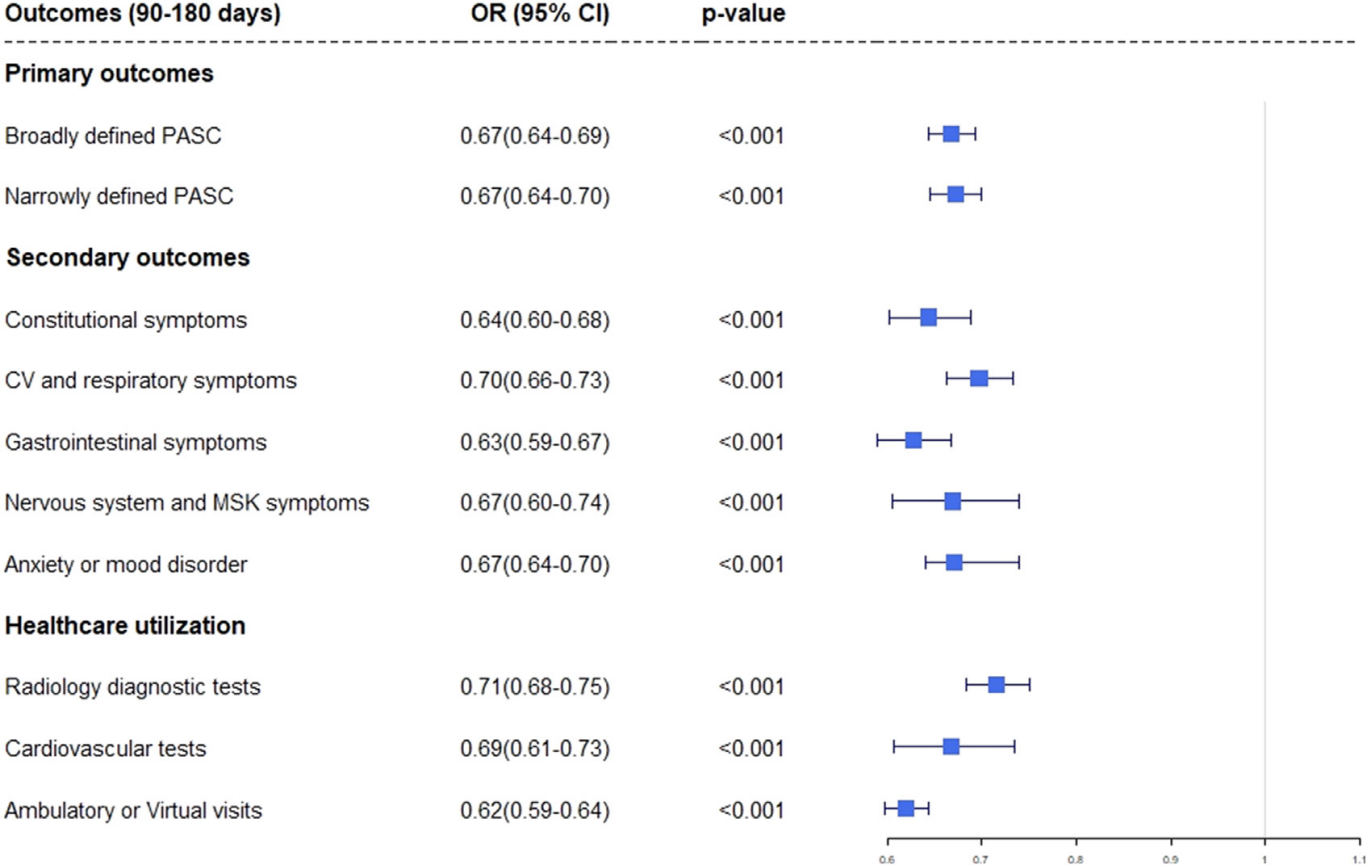
**Forest Plot of Postacute Sequelae of SARS-CoV-2 Infection Outcomes at 90 to 180 Days After the Index COVID-19 Infection** This Forest plot depicts the primary and secondary outcomes within 90 to 180 days after the diagnosis of COVID-19. CV = cardiovascular; MSK = musculoskeletal; PASC = postacute sequelae of SARS-CoV-2 infection.

Patients who had been treated with NMV-r within 5 days of infection with COVID-19 had lower odds and reduced risk of having cardiovascular and respiratory symptoms (OR: 0.70, 95% CI: 0.66-0.73, *P* < 0.001); gastrointestinal symptoms (OR: 0.63, 95% CI 0.59-0.67, *P* < 0.001); anxiety/mood disorders (OR: 0.67, 95% CI: 0.64-0.70, *P* < 0.001); and lower health care utilization including radiology and cardiovascular diagnostic tests and ambulatory/virtual visits between 90 and 180 days postinfection. This has been depicted in Table 3 as well as Figure 3.

**Table 3:** Postacute Sequelae of SARS-CoV-2 Infection Outcomes at 90 to 180 Days After Index COVID-19 Infection

						E Values		
Outcomes (90-180 d)	NMV-r Cohort (n = 26,593)	Non-NMV-r Cohort (n = 26,593)^a^	Risk Difference (95% CI)	Relative Risk Reduction (95% CI)	OR (95% CI)	OR	Lower CI for OR	*P* Value
Primary outcome								
Broadly defined PASC	6,692 (25.1%)	8,910 (33.5%)	−0.083 (−0.091 to −0.076)	25% (23%-27%)	0.667 (0.643–0.693)	1.75	1.8	<0.001
Narrowly defined PASC	5,121 (19.2%)	6,964 (26.2%)	−0.069 (−0.076 to −0.062)	26% (24%-29%)	0.672 (0.645–0.700)	1.74	1.8	<0.001
Secondary outcomes								
Individual symptoms								
Constitutional symptoms	1,555 (5.84%)	2,341 (8.8%)	−0.030 (−0.034 to −0.025)	33% (29%-37%)	0.643 (0.602–0.688)	2.48	2.71	<0.001
CV and respiratory symptoms	3,062 (11.5%)	4,182 (15.7%)	−0.042 (−0.048 to −0.036)	27% (23%-30%)	0.697 (0.663–0.733)	2.22	2.38	<0.001
Gastrointestinal symptoms	1,737 (6.5%)	2,666 (10%)	−0.035 (−0.040 to −0.030)	35% (31%-38%)	0.627 (0.589–0.668)	2.57	2.79	<0.001
Nervous system and MSK symptoms	661 (2.48%)	976 (3.6%)	−0.012 (−0.015 to −0.009)	32% (25%-38%)	0.669 (0.605–0.740)	2.35	2.69	<0.001
Anxiety/mood disorder	3,478 (13.1%)	4,870 (18.3%)	−0.052 (−0.059 to −0.046)	28% (25%-31%)	0.671 (0.640–0.704)	2.35	2.5	<0.001
Healthcare Utilization								
Radiology diagnostic tests	3,888 (14.6%)	5,131 (19.2%)	−0.047 (−0.053 to −0.040)	24% (21%-27%)	0.716 (0.684–0.750)	2.14	2.28	<0.001
CV tests (echocardiogram and ambulatory rhythm monitors)	725 (2.7%)	1,071 (4%)	−0.013 (−0.016 to −0.010)	32% (26%-38%)	0.668 (0.607–0.735)	2.36	2.68	<0.001
AMB/Virtual visit	15,921 (59.8%)	18,782 (70.6%)	−0.108 (−0.116 to −0.100)	15% (14%-16%)	0.620 (0.598–0.643)	1.86	1.91	<0.001

Values are n (%) unless otherwise indicated.

AMB = ambulatory; CV = cardiovascular; ER = emergency room; MSK = musculoskeletal.

a After propensity score matching.

## Discussion

Our study uniquely demonstrates significant risk reduction (15%-26% relative risk reduction) in PASC burden in high-risk patients with pre-existing CVD receiving NMV-r for the treatment of acute COVID-19. This relative decrease in reported symptoms with treatment was observed using broad and narrow definitions of PASC between 30 to 90 days and 90 to 180 days after index infection. The cardiovascular, respiratory, gastrointestinal, constitutional, musculoskeletal, nervous system, and mood/cognitive disorder-related symptoms were recorded less frequently among those prescribed NMV-r than those not treated with NMV-r (Figures 2 and 3, Central Illustration). Overall health care utilization, including radiology and cardiovascular diagnostic tests and ambulatory/virtual visits, was lower among those who received NMV-r.

**Central Illustration:**
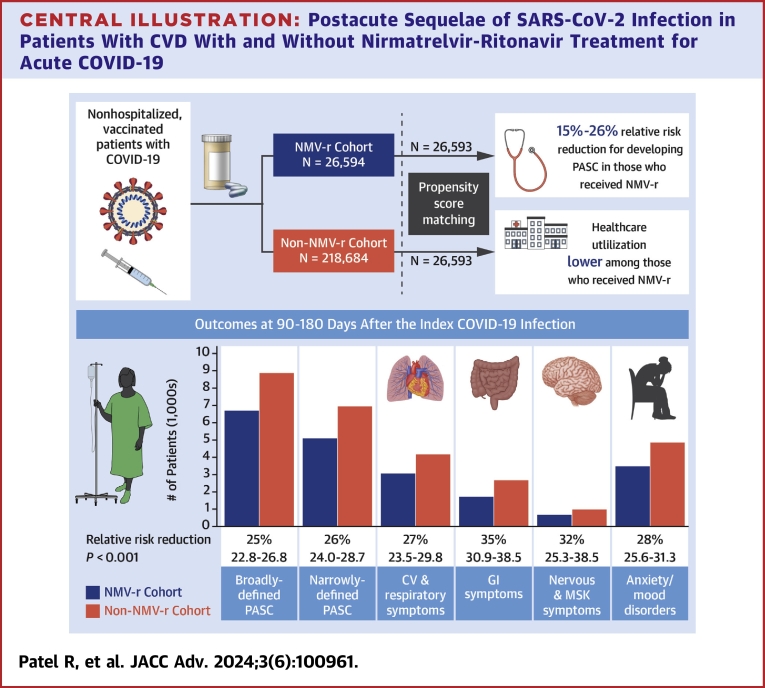
**Postacute Sequelae of SARS-CoV-2 Infection in Patients With CVD With and Without Nirmatrelvir-Ritonavir Treatment for Acute COVID-19** CVD = cardiovascular diseases; NMV-r = nirmatrelvir plus ritonavir; PASC = postacute sequelae of SARS-CoV-2 infection.

A strength of this analysis is that it included only vaccinated patients who contracted COVID-19 when the Omicron variant was widely circulating, making it reflective of the current status of both the population’s high level of vaccine- or infection-induced immunity and the potential for Omicron to cause less severe disease than prior variants.

There are discrepancies in the definition of PASC, as evident from heterogeneous definitions by the National Institute of Health’s Researching COVID to Enhance Recovery Initiative and the World Health Organization.^17^^,^^18^^,^^22^ Therefore, it is important to clarify that our study does not specifically focus on diagnosing PASC but utilizes the reporting of PASC as an outcome measure. The symptoms in our analysis are similar to a recent study defining PASC^6^; however, we did not use the PASC scoring system. We further evaluated the reporting of these symptoms using 2 separate timelines: 1) 30 to 90 days postinfection; and 2) 90 to 180 days after the index diagnosis of COVID-19.

Patients with pre-existing CVD are at a higher risk for developing PASC.^5^ Quantitative and qualitative studies have demonstrated the complexity of diagnosing PASC in this subset of patients. One of the reasons is the similarity in the symptomatology of CVDs and PASC. As it is essential to differentiate the symptoms' etiology, it leads to higher diagnostic testing and eventually increased overall health care utilization. Additionally, commonality in symptoms may lead to a delay in diagnosis or misdiagnosis of PASC and CVD. Our findings suggest that using NMV-r in treating acute COVID-19 among patients with pre-existing CVD may help mitigate PASC, reduce the risk of CV complications, and reduce downstream health care utilization as shown in the Central Illustration.

While the pathophysiology of PASC remains uncertain, there seems to be some correlation between initial disease severity and the likelihood of developing PASC, though even some people with mild COVID-19 can experience prolonged and disabling symptoms.^23^ There is evidence that vaccination reduces the incidence of PASC by 15 to 50%,^24^ and the incidence also appears to be lower with Omicron compared with infection with earlier variants.^25^ Moreover, there is growing evidence of the persistence of viral particles throughout the human body after resolution of acute SARS-CoV-2 infection.^14^ A small study demonstrated that patients diagnosed with PASC had evidence of persistent SARS-CoV-2 antigen detected in blood samples,^14^ thought to be one of the potential mechanisms of the pathophysiology of PASC.^23^ This raises the possibility that potential interventions that reduce viral replication or the duration of acute disease will reduce the risk of PASC.

Our study is the first and largest to demonstrate the incremental benefit of NMV-r in reducing the burden of PASC among vaccinated patients with pre-existing CVD who developed COVID-19. This potentially has multiple downstream positive impacts and will guide therapeutic interventions for COVID-19 in patients with CVD. Our results are similar to those reported in the study by the Department of Veterans Affairs.^11^ However, in contrast with that study, our definition of PASC-associated symptoms is more diverse. While all patients in our study had 1 or more pre-existing CVD or risk factors, only 30% to 40% of patients in the Department of Veterans Affairs study had CVD or risk factors. Moreover, our patient population includes only those vaccinated, a much higher proportion of women, and a longer follow-up period. The inclusion of more women in our study is vital, as PASC appears to be more common in women.^5^

As per the reports, NMV-r for Treatment of Long Covid prospective clinical trial has recently halted further enrollment in light of inconclusive evidence (NCT05576662, NCT05595369). However, it is important to note that this was a trial examining the role of NMV-r in all adult patients (with or without CVD) presenting with PASC in contrast to our study, which examines the impact of NMV-r in reducing PASC when used for the treatment of acute COVID-19 and includes only vaccinated adult patients with 1 or more pre-existing CVD. It is a known fact that NMV-r, like other antiviral agents, is most effective when used early on, and the observed striking differences between these 2 studies are possibly due to the timeline of the treatment and selective patient population in our study with pre-existing CVD, a known risk factor for adverse outcomes. Importantly, patients with CVD are more likely to receive NMV-r, it is crucial to be aware of significant drug-drug interactions while prescribing NMV-r.^26^

### Study Limitations

Despite these observed favorable associations between NMV-r and reduced long-term symptoms, our data cannot prove that antiviral treatment reduces the incidence of PASC. As in many observational studies, the group receiving treatment differed significantly from those not receiving treatment, differences that could influence the results independent of the treatment they received. These biases could increase the risk of PASC in those accessing treatment and, conversely, decrease it. More severe symptoms could trigger reaching out for treatment and subsequent health care utilization, making PASC more likely in those receiving treatment. Conversely, patients with PASC who did not seek health care services might have been excluded from the analysis. By contrast, patients who initially did not seek treatment but then later progressed may have fallen out of the 5 days from symptom onset treatment eligibility, rendering them unable to get NMV-r. While we controlled for differing baseline characteristics using propensity matching, residual confounding remains a significant potential limitation given the study's observational nature. Furthermore, as no dates were associated with the codes for the broad and narrow definitions of PASC, the granular details of the timing of symptoms, positive tests, and subsequent healthcare utilization were not generated, negating the possibility of an appropriate sensitivity analysis testing.

Several additional limitations intrinsic to the study design and the condition deserve mention. The retrospective design relied on the accuracy of the data entered in the EHR by point-of-care clinical providers; hence, despite separately analyzing data for broad and narrow-based PASC, our definition may have lacked precision and may have been too inclusive. An intrinsic challenge in the study of PASC is the highly variable nature of the condition, which may involve multiple organ systems and a vast range of clinical severity, from mild fatigue to complete disability and no established diagnostic test or tests. Another limitation inherent to any large retrospective study like ours also includes the possibility of subjective variation in EHR recording data, which can impact the timing of testing, diagnoses, and treatment dates.

Moreover, our study included patients older than 18 years of age, limiting the generalizability of results to children >12 years of age who can develop PASC and are eligible for the medication.

Limitations notwithstanding, our study supports the hypothesis that treating the acute infection with NMV-r may reduce the symptoms associated with PASC. It furthermore should reassure prescribers that treatment does not *increase* this risk, a potential concern given the widely observed viral and symptomatic rebound phenomenon. While a more accurate assessment of the effect of NMV-r treatment on the incidence of PASC would optimally come from the original placebo-controlled trials, EPIC-HR^7^ and EPIC-SR (NCT05011513), these analyses have not been conducted or reported. Studies of long-term outcomes are planned in more recent randomized trials, including COVID-OUT,^27^ ACTIV-6,^28^ and PANORAMIC.^29^

Lastly, drug-drug interactions, especially with cardiovascular drugs are crucial to consider in this population,^26^ however, given the retrospective nature of the study and the limitations of the database, it was not feasible to assess for these interactions or comment on interruption in the medications or alteration in dosages.

## Conclusions

In a retrospective analysis looking at long-term outcomes after diagnosis of COVID-19 in patients with pre-existing CVD, these data suggest an association between treatment with NMV-r and a reduced incidence of the symptoms commonly reported with PASC. As the case severity of COVID-19 is much lower than when the pandemic first started, outcomes aside from hospitalization and death must be rigorously analyzed, particularly in this subset of high-risk patients. As such, prospective studies of the potential effect of antiviral therapy on the incidence of PASC are urgently needed. But, pending these data, our study does support the use of NMV-r to decrease even potential long-term complications of COVID-19, an important observation given the continued low rate of prescribing for this medication.

## Funding support and author disclosures

The authors have reported that they have no relationships relevant to the contents of this paper to disclose.

PERSPECTIVES **COMPETENCY IN MEDICAL KNOWLEDGE:** In a retrospective analysis, there is a significant risk reduction in PASC burden in high-risk patients with pre-existing CVD receiving NMV-r for the treatment of acute COVID-19 suggesting an association between treatment with NMV-r and a reduced incidence of the symptoms commonly reported with PASC.

**TRANSLATIONAL OUTLOOK:** Prospective studies in the form of randomized controlled trials are urgently needed to understand the potential effect of antiviral therapy on the incidence of PASC.
