# Supplementary material for: Nirmatrelvir-Ritonavir for Acute COVID-19 in Patients With Cardiovascular Disease and Postacute Sequelae of SARS-CoV-2 Infection
Source: JACC Adv. 2024 Jun 26;3(6):100961. doi: 10.1016/j.jacadv.2024.100961 (PMC11286995; doi:10.1016/j.jacadv.2024.100961)
Supplement: Supplemental Appendix [file mmc1.docx]

Supplemental Appendix

## Cohorts definition

This section lists all terms used in the definitions of the two cohorts.

### Query Criteria for Cohort 1 (CVD, Received NMV-r)

This query was run on the network Research with 80 HCO(s) queried and 80 HCO(s) responded. A total of 34 provider(s) responded with patients. The final cohort included 26,594 patients who matched the query criteria listed in the table below. For the text representation of the query criteria please see Appendix A.

|  | | | | | |
| --- | --- | --- | --- | --- | --- |
| Ungrouped terms | | | | | |
|  | must have |  | demographics | Age | Age (at least 18 years (most recent occurrence)) |
|  |  | and any of | medication | NLM:VA:CV100 | BETA BLOCKERS/RELATED |
|  |  |  | medication | NLM:VA:CV150 | ALPHA BLOCKERS/RELATED |
|  |  |  | medication | NLM:VA:CV200 | CALCIUM CHANNEL BLOCKERS |
|  |  |  | medication | NLM:VA:CV250 | ANTIANGINALS |
|  |  |  | medication | NLM:VA:CV350 | ANTILIPEMIC AGENTS |
|  |  |  | medication | NLM:VA:CV400 | ANTIHYPERTENSIVE COMBINATIONS |
|  |  |  | medication | NLM:VA:CV500 | PERIPHERAL VASODILATORS |
|  |  |  | medication | NLM:VA:CV700 | DIURETICS |
|  |  |  | medication | NLM:VA:CV800 | ACE INHIBITORS |
|  |  |  | medication | NLM:VA:CV805 | ANGIOTENSIN II INHIBITOR |
|  |  |  | diagnosis | UMLS:ICD10CM:I10-I16 | Hypertensive diseases |
|  |  |  | diagnosis | UMLS:ICD10CM:I20-I25 | Ischemic heart diseases |
|  |  |  | diagnosis | UMLS:ICD10CM:I26-I28 | Pulmonary heart disease and diseases of pulmonary circulation |
|  |  |  | diagnosis | UMLS:ICD10CM:I60-I69 | Cerebrovascular diseases |
|  |  |  | diagnosis | UMLS:ICD10CM:E11 | Type 2 diabetes mellitus |
|  |  |  | diagnosis | UMLS:ICD10CM:I42 | Cardiomyopathy |
|  |  |  | diagnosis | UMLS:ICD10CM:I43 | Cardiomyopathy in diseases classified elsewhere |
|  |  |  | diagnosis | UMLS:ICD10CM:I47 | Paroxysmal tachycardia |
|  |  |  | diagnosis | UMLS:ICD10CM:I48 | Atrial fibrillation and flutter |
|  |  |  | diagnosis | UMLS:ICD10CM:I49 | Other cardiac arrhythmias |
|  |  |  | diagnosis | UMLS:ICD10CM:I50 | Heart failure |
| Group 1 | | | | | |
|  | **Group 1A Vaccine** | | | | |
|  | must have | any of | procedure | UMLS:CPT:91300 | Severe acute respiratory syndrome coronavirus 2 (SARS-CoV-2) (Coronavirus disease [COVID-19]) vaccine, mRNA-LNP, spike protein, preservative free, 30 mcg/0.3mL dosage, diluent reconstituted, for intramuscular use |
|  |  |  | procedure | UMLS:CPT:91300 | Severe acute respiratory syndrome coronavirus 2 (SARS-CoV-2) (Coronavirus disease [COVID-19]) vaccine, mRNA-LNP, spike protein, preservative free, 30 mcg/0.3mL dosage, diluent reconstituted, for intramuscular use |
|  |  |  | procedure | UMLS:CPT:0001A | Immunization administration by intramuscular injection of severe acute respiratory syndrome coronavirus 2 (SARS-CoV-2) (Coronavirus disease [COVID-19]) vaccine, mRNA-LNP, spike protein, preservative free, 30 mcg/0.3mL dosage, diluent reconstituted; first dose |
|  |  |  | procedure | UMLS:CPT:0002A | Immunization administration by intramuscular injection of severe acute respiratory syndrome coronavirus 2 (SARS-CoV-2) (Coronavirus disease [COVID-19]) vaccine, mRNA-LNP, spike protein, preservative free, 30 mcg/0.3mL dosage, diluent reconstituted; second dose |
|  |  |  | procedure | UMLS:CPT:91301 | Severe acute respiratory syndrome coronavirus 2 (SARS-CoV-2) (Coronavirus disease [COVID-19]) vaccine, mRNA-LNP, spike protein, preservative free, 100 mcg/0.5mL dosage, for intramuscular use |
|  |  |  | procedure | UMLS:CPT:0011A | Immunization administration by intramuscular injection of severe acute respiratory syndrome coronavirus 2 (SARS-CoV-2) (Coronavirus disease [COVID-19]) vaccine, mRNA-LNP, spike protein, preservative free, 100 mcg/0.5mL dosage; first dose |
|  |  |  | procedure | UMLS:CPT:0012A | Immunization administration by intramuscular injection of severe acute respiratory syndrome coronavirus 2 (SARS-CoV-2) (Coronavirus disease [COVID-19]) vaccine, mRNA-LNP, spike protein, preservative free, 100 mcg/0.5mL dosage; second dose |
|  |  |  | procedure | UMLS:CPT:91303 | Severe acute respiratory syndrome coronavirus 2 (SARS-CoV-2) (coronavirus disease [COVID-19]) vaccine, DNA, spike protein, adenovirus type 26 (Ad26) vector, preservative free, 5x10^10 viral particles/0.5mL dosage, for intramuscular use |
|  |  |  | procedure | UMLS:CPT:0031A | Immunization administration by intramuscular injection of severe acute respiratory syndrome coronavirus 2 (SARS-CoV-2) (coronavirus disease [COVID-19]) vaccine, DNA, spike protein, adenovirus type 26 (Ad26) vector, preservative free, 5x10^10 viral particles/0.5mL dosage, single dose |
|  |  |  | procedure | UMLS:ICD10PCS:XW023S6 | Introduction of COVID-19 Vaccine Dose 1 into Muscle, Percutaneous Approach, New Technology Group 6 |
|  |  |  | procedure | UMLS:ICD10PCS:XW023T6 | Introduction of COVID-19 Vaccine Dose 2 into Muscle, Percutaneous Approach, New Technology Group 6 |
|  |  |  | procedure | UMLS:ICD10PCS:XW013S6 | Introduction of COVID-19 Vaccine Dose 1 into Subcutaneous Tissue, Percutaneous Approach, New Technology Group 6 |
|  |  |  | procedure | UMLS:ICD10PCS:XW013U6 | Introduction of COVID-19 Vaccine into Subcutaneous Tissue, Percutaneous Approach, New Technology Group 6 |
|  |  |  | procedure | UMLS:CPT:0021A | Immunization administration by intramuscular injection of severe acute respiratory syndrome coronavirus 2 (SARS-CoV-2) (coronavirus disease [COVID-19]) vaccine, DNA, spike protein, chimpanzee adenovirus Oxford 1 (ChAdOx1) vector, preservative free, 5x10^10 viral particles/0.5mL dosage; first dose |
|  |  |  | procedure | UMLS:CPT:0022A | Immunization administration by intramuscular injection of severe acute respiratory syndrome coronavirus 2 (SARS-CoV-2) (coronavirus disease [COVID-19]) vaccine, DNA, spike protein, chimpanzee adenovirus Oxford 1 (ChAdOx1) vector, preservative free, 5x10^10 viral particles/0.5mL dosage; second dose |
|  |  |  | procedure | UMLS:CPT:91302 | Severe acute respiratory syndrome coronavirus 2 (SARS-CoV-2) (coronavirus disease [COVID-19]) vaccine, DNA, spike protein, chimpanzee adenovirus Oxford 1 (ChAdOx1) vector, preservative free, 5x10^10 viral particles/0.5mL dosage, for intramuscular use |
|  |  |  | procedure | UMLS:HCPCS:U0003 | Infectious agent detection by nucleic acid (dna or rna); severe acute respiratory syndrome coronavirus 2 (sars-cov-2) (coronavirus disease [covid-19]), amplified probe technique, making use of high throughput technologies as described by cms-2020-01-r |
|  |  |  | medication | NLM:CVX:213 | SARS-CoV-2 (COVID-19) Vaccine |
|  |  |  | procedure | UMLS:CPT:0003A | Immunization administration by intramuscular injection of severe acute respiratory syndrome coronavirus 2 (SARS-CoV-2) (Coronavirus disease [COVID-19]) vaccine, mRNA-LNP, spike protein, preservative free, 30 mcg/0.3mL dosage, diluent reconstituted; third dose |
|  |  |  | medication | NLM:RXNORM:2479831 | SARS-COV-2 (COVID-19) vaccine, vector non-replicating |
|  |  |  | procedure | UMLS:CPT:0071A | Immunization administration by intramuscular injection of severe acute respiratory syndrome coronavirus 2 (SARS-CoV-2) (coronavirus disease [COVID-19]) vaccine, mRNA-LNP, spike protein, preservative free, 10 mcg/0.2 mL dosage, diluent reconstituted, tris-sucrose formulation; first dose |
|  |  |  | procedure | UMLS:CPT:91306 | Severe acute respiratory syndrome coronavirus 2 (SARS-CoV-2) (coronavirus disease [COVID-19]) vaccine, mRNA-LNP, spike protein, preservative free, 50 mcg/0.25 mL dosage, for intramuscular use |
|  |  |  | procedure | UMLS:CPT:0064A | Immunization administration by intramuscular injection of severe acute respiratory syndrome coronavirus 2 (SARS-CoV-2) (coronavirus disease [COVID-19]) vaccine, mRNA-LNP, spike protein, preservative free, 50 mcg/0.25 mL dosage, booster dose |
|  |  |  | procedure | UMLS:CPT:0072A | Immunization administration by intramuscular injection of severe acute respiratory syndrome coronavirus 2 (SARS-CoV-2) (coronavirus disease [COVID-19]) vaccine, mRNA-LNP, spike protein, preservative free, 10 mcg/0.2 mL dosage, diluent reconstituted, tris-sucrose formulation; second dose |
|  |  |  | procedure | UMLS:CPT:0013A | Immunization administration by intramuscular injection of severe acute respiratory syndrome coronavirus 2 (SARS-CoV-2) (coronavirus disease [COVID-19]) vaccine, mRNA-LNP, spike protein, preservative free, 100 mcg/0.5 mL dosage; third dose |
|  |  |  | procedure | UMLS:CPT:91305 | Severe acute respiratory syndrome coronavirus 2 (SARS-CoV-2) (coronavirus disease [COVID-19]) vaccine, mRNA-LNP, spike protein, preservative free, 30 mcg/0.3 mL dosage, tris-sucrose formulation, for intramuscular use |
|  |  |  | procedure | UMLS:ICD10PCS:XW023U6 | Introduction of COVID-19 Vaccine into Muscle, Percutaneous Approach, New Technology Group 6 |
|  |  |  | procedure | UMLS:CPT:0054A | Immunization administration by intramuscular injection of severe acute respiratory syndrome coronavirus 2 (SARS-CoV-2) (coronavirus disease [COVID-19]) vaccine, mRNA-LNP, spike protein, preservative free, 30 mcg/0.3 mL dosage, tris-sucrose formulation; booster dose |
|  |  |  | procedure | UMLS:CPT:0034A | Immunization administration by intramuscular injection of severe acute respiratory syndrome coronavirus 2 (SARS-CoV-2) (coronavirus disease [COVID-19]) vaccine, DNA, spike protein, adenovirus type 26 (Ad26) vector, preservative free, 5x10^10 viral particles/0.5 mL dosage; booster dose |
|  |  |  | procedure | UMLS:CPT:0051A | Immunization administration by intramuscular injection of severe acute respiratory syndrome coronavirus 2 (SARS-CoV-2) (coronavirus disease [COVID-19]) vaccine, mRNA-LNP, spike protein, preservative free, 30 mcg/0.3 mL dosage, tris-sucrose formulation; first dose |
|  |  |  | procedure | UMLS:CPT:0053A | Immunization administration by intramuscular injection of severe acute respiratory syndrome coronavirus 2 (SARS-CoV-2) (coronavirus disease [COVID-19]) vaccine, mRNA-LNP, spike protein, preservative free, 30 mcg/0.3 mL dosage, tris-sucrose formulation; third dose |
|  |  |  | procedure | UMLS:CPT:0052A | Immunization administration by intramuscular injection of severe acute respiratory syndrome coronavirus 2 (SARS-CoV-2) (coronavirus disease [COVID-19]) vaccine, mRNA-LNP, spike protein, preservative free, 30 mcg/0.3 mL dosage, tris-sucrose formulation; second dose |
|  |  |  | procedure | UMLS:ICD10PCS:XW013T6 | Introduction of COVID-19 Vaccine Dose 2 into Subcutaneous Tissue, Percutaneous Approach, New Technology Group 6 |
|  | date constraint | | The terms in this group occurred on or before Dec 31, 2022 | | |
|  | event relationship | | Any instance of Group 1B occurred at least 1 month after any instance of Vaccine | | |
|  | **Group 1B** | | | | |
|  | must have | any of | laboratory | TNX:9088 | SARS coronavirus 2 and related RNA [Presence] (labResult: Positive) |
|  |  |  | laboratory | UMLS:LNC:94500-6 | SARS-CoV-2 (COVID19) RNA [Presence] in Respiratory specimen by NAA with probe detection (labResult: Positive) |
|  |  |  | laboratory | TNX:9089 | SARS coronavirus 2 IgG IgM Ab [Presence] in Serum or Plasma (labResult: Positive) |
|  |  |  | laboratory | TNX:9088 | SARS coronavirus 2 and related RNA [Presence] (labResult: Positive) |
|  |  |  | diagnosis | UMLS:ICD10CM:U07.1 | COVID-19 |
|  |  |  | laboratory | TNX:9088 | SARS coronavirus 2 and related RNA [Presence] (labResult: Positive) |
|  |  |  | laboratory | UMLS:LNC:94500-6 | SARS-CoV-2 (COVID19) RNA [Presence] in Respiratory specimen by NAA with probe detection (labResult: Positive) |
|  |  |  | laboratory | UMLS:LNC:94309-2 | SARS-CoV-2 (COVID-19) RNA [Presence] in Specimen by NAA with probe detection (labResult: Positive) |
|  |  |  | laboratory | UMLS:LNC:94534-5 | SARS-CoV-2 (COVID-19) RdRp gene [Presence] in Respiratory specimen by NAA with probe detection (labResult: Positive) |
|  |  |  | laboratory | UMLS:LNC:94565-9 | SARS-CoV-2 (COVID-19) RNA [Presence] in Nasopharynx by NAA with non-probe detection (labResult: Positive) |
|  |  |  | laboratory | UMLS:LNC:94316-7 | SARS-CoV-2 (COVID-19) N gene [Presence] in Specimen by NAA with probe detection (labResult: Positive) |
|  |  |  | laboratory | UMLS:LNC:95209-3 | SARS-CoV+SARS-CoV-2 (COVID-19) Ag [Presence] in Respiratory specimen by Rapid immunoassay (labResult: Positive) |
|  |  |  | laboratory | UMLS:LNC:94559-2 | SARS-CoV-2 (COVID-19) ORF1ab region [Presence] in Respiratory specimen by NAA with probe detection (labResult: Positive) |
|  |  |  | laboratory | UMLS:LNC:94759-8 | SARS-CoV-2 (COVID-19) RNA [Presence] in Nasopharynx by NAA with probe detection (labResult: Positive) |
|  |  |  | laboratory | UMLS:LNC:94558-4 | SARS-CoV-2 (COVID-19) Ag [Presence] in Respiratory specimen by Rapid immunoassay (labResult: Positive) |
|  |  |  | laboratory | UMLS:LNC:95608-6 | SARS-CoV-2 (COVID-19) RNA [Presence] in Respiratory specimen by NAA with non-probe detection (labResult: Positive) |
|  |  |  | laboratory | UMLS:LNC:94508-9 | SARS-CoV-2 (COVID-19) IgM Ab [Presence] in Serum, Plasma or Blood by Rapid immunoassay (labResult: Positive) |
|  |  |  | laboratory | UMLS:LNC:94763-0 | SARS-CoV-2 (COVID-19) [Presence] in Specimen by Organism specific culture (labResult: Positive) |
|  |  |  | laboratory | UMLS:LNC:96119-3 | SARS-CoV-2 (COVID-19) Ag [Presence] in Upper respiratory specimen by Immunoassay (labResult: Positive) |
|  |  |  | laboratory | UMLS:LNC:94533-7 | SARS-CoV-2 (COVID-19) N gene [Presence] in Respiratory specimen by NAA with probe detection (labResult: Positive) |
|  |  |  | laboratory | UMLS:LNC:94564-2 | SARS-CoV-2 (COVID-19) IgM Ab [Presence] in Serum or Plasma by Immunoassay (labResult: Positive) |
|  |  |  | laboratory | UMLS:LNC:95406-5 | SARS-CoV-2 (COVID-19) RNA [Presence] in Nose by NAA with probe detection (labResult: Positive) |
|  |  |  | laboratory | UMLS:LNC:94758-0 | SARS-related coronavirus E gene [Presence] in Respiratory specimen by NAA with probe detection (labResult: Positive) |
|  |  |  | laboratory | UMLS:LNC:96763-8 | SARS-CoV-2 (COVID-19) E gene [Presence] in Respiratory specimen by NAA with probe detection (labResult: Positive) |
|  |  |  | laboratory | UMLS:LNC:94502-2 | SARS-related coronavirus RNA [Presence] in Respiratory specimen by NAA with probe detection (labResult: Positive) |
|  |  |  | laboratory | UMLS:LNC:94760-6 | SARS-CoV-2 (COVID-19) N gene [Presence] in Nasopharynx by NAA with probe detection (labResult: Positive) |
|  |  |  | laboratory | UMLS:LNC:94314-2 | SARS-CoV-2 (COVID-19) RdRp gene [Presence] in Specimen by NAA with probe detection (labResult: Positive) |
|  |  |  | laboratory | UMLS:LNC:97097-0 | SARS-CoV-2 (COVID-19) Ag [Presence] in Upper respiratory specimen by Rapid immunoassay (labResult: Positive) |
|  |  |  | laboratory | UMLS:LNC:94647-5 | SARS-related coronavirus RNA [Presence] in Specimen by NAA with probe detection (labResult: Positive) |
|  |  |  | laboratory | UMLS:LNC:94845-5 | SARS-CoV-2 (COVID-19) RNA [Presence] in Saliva (oral fluid) by NAA with probe detection (labResult: Positive) |
|  |  |  | laboratory | UMLS:LNC:95409-9 | SARS-CoV-2 (COVID-19) N gene [Presence] in Nose by NAA with probe detection (labResult: Positive) |
|  |  |  | diagnosis | UMLS:ICD10CM:U07.1 | COVID-19 |
| Group 2 | | | | | |
|  | **Group 2A** | | | | |
|  | must have | any of | laboratory | TNX:9088 | SARS coronavirus 2 and related RNA [Presence] (labResult: Positive) |
|  |  |  | laboratory | UMLS:LNC:94500-6 | SARS-CoV-2 (COVID19) RNA [Presence] in Respiratory specimen by NAA with probe detection (labResult: Positive) |
|  |  |  | laboratory | TNX:9089 | SARS coronavirus 2 IgG IgM Ab [Presence] in Serum or Plasma (labResult: Positive) |
|  |  |  | laboratory | TNX:9088 | SARS coronavirus 2 and related RNA [Presence] (labResult: Positive) |
|  |  |  | diagnosis | UMLS:ICD10CM:U07.1 | COVID-19 |
|  |  |  | laboratory | TNX:9088 | SARS coronavirus 2 and related RNA [Presence] (labResult: Positive) |
|  |  |  | laboratory | UMLS:LNC:94500-6 | SARS-CoV-2 (COVID19) RNA [Presence] in Respiratory specimen by NAA with probe detection (labResult: Positive) |
|  |  |  | laboratory | UMLS:LNC:94309-2 | SARS-CoV-2 (COVID-19) RNA [Presence] in Specimen by NAA with probe detection (labResult: Positive) |
|  |  |  | laboratory | UMLS:LNC:94534-5 | SARS-CoV-2 (COVID-19) RdRp gene [Presence] in Respiratory specimen by NAA with probe detection (labResult: Positive) |
|  |  |  | laboratory | UMLS:LNC:94565-9 | SARS-CoV-2 (COVID-19) RNA [Presence] in Nasopharynx by NAA with non-probe detection (labResult: Positive) |
|  |  |  | laboratory | UMLS:LNC:94316-7 | SARS-CoV-2 (COVID-19) N gene [Presence] in Specimen by NAA with probe detection (labResult: Positive) |
|  |  |  | laboratory | UMLS:LNC:95209-3 | SARS-CoV+SARS-CoV-2 (COVID-19) Ag [Presence] in Respiratory specimen by Rapid immunoassay (labResult: Positive) |
|  |  |  | laboratory | UMLS:LNC:94559-2 | SARS-CoV-2 (COVID-19) ORF1ab region [Presence] in Respiratory specimen by NAA with probe detection (labResult: Positive) |
|  |  |  | laboratory | UMLS:LNC:94759-8 | SARS-CoV-2 (COVID-19) RNA [Presence] in Nasopharynx by NAA with probe detection (labResult: Positive) |
|  |  |  | laboratory | UMLS:LNC:94558-4 | SARS-CoV-2 (COVID-19) Ag [Presence] in Respiratory specimen by Rapid immunoassay (labResult: Positive) |
|  |  |  | laboratory | UMLS:LNC:95608-6 | SARS-CoV-2 (COVID-19) RNA [Presence] in Respiratory specimen by NAA with non-probe detection (labResult: Positive) |
|  |  |  | laboratory | UMLS:LNC:94508-9 | SARS-CoV-2 (COVID-19) IgM Ab [Presence] in Serum, Plasma or Blood by Rapid immunoassay (labResult: Positive) |
|  |  |  | laboratory | UMLS:LNC:94763-0 | SARS-CoV-2 (COVID-19) [Presence] in Specimen by Organism specific culture (labResult: Positive) |
|  |  |  | laboratory | UMLS:LNC:96119-3 | SARS-CoV-2 (COVID-19) Ag [Presence] in Upper respiratory specimen by Immunoassay (labResult: Positive) |
|  |  |  | laboratory | UMLS:LNC:94533-7 | SARS-CoV-2 (COVID-19) N gene [Presence] in Respiratory specimen by NAA with probe detection (labResult: Positive) |
|  |  |  | laboratory | UMLS:LNC:94564-2 | SARS-CoV-2 (COVID-19) IgM Ab [Presence] in Serum or Plasma by Immunoassay (labResult: Positive) |
|  |  |  | laboratory | UMLS:LNC:95406-5 | SARS-CoV-2 (COVID-19) RNA [Presence] in Nose by NAA with probe detection (labResult: Positive) |
|  |  |  | laboratory | UMLS:LNC:94758-0 | SARS-related coronavirus E gene [Presence] in Respiratory specimen by NAA with probe detection (labResult: Positive) |
|  |  |  | laboratory | UMLS:LNC:96763-8 | SARS-CoV-2 (COVID-19) E gene [Presence] in Respiratory specimen by NAA with probe detection (labResult: Positive) |
|  |  |  | laboratory | UMLS:LNC:94502-2 | SARS-related coronavirus RNA [Presence] in Respiratory specimen by NAA with probe detection (labResult: Positive) |
|  |  |  | laboratory | UMLS:LNC:94760-6 | SARS-CoV-2 (COVID-19) N gene [Presence] in Nasopharynx by NAA with probe detection (labResult: Positive) |
|  |  |  | laboratory | UMLS:LNC:94314-2 | SARS-CoV-2 (COVID-19) RdRp gene [Presence] in Specimen by NAA with probe detection (labResult: Positive) |
|  |  |  | laboratory | UMLS:LNC:97097-0 | SARS-CoV-2 (COVID-19) Ag [Presence] in Upper respiratory specimen by Rapid immunoassay (labResult: Positive) |
|  |  |  | laboratory | UMLS:LNC:94647-5 | SARS-related coronavirus RNA [Presence] in Specimen by NAA with probe detection (labResult: Positive) |
|  |  |  | laboratory | UMLS:LNC:94845-5 | SARS-CoV-2 (COVID-19) RNA [Presence] in Saliva (oral fluid) by NAA with probe detection (labResult: Positive) |
|  |  |  | laboratory | UMLS:LNC:95409-9 | SARS-CoV-2 (COVID-19) N gene [Presence] in Nose by NAA with probe detection (labResult: Positive) |
|  |  |  | diagnosis | UMLS:ICD10CM:U07.1 | COVID-19 |
|  | date constraint | | The terms in this group occurred at any time | | |
|  | event relationship | | Any instance of Group 2B occurred within 5 days on or after any instance of Group 2A | | |
|  | **Group 2B** | | | | |
|  | must have |  | medication | NLM:RXNORM:85762 | ritonavir |
|  |  | and | medication | NLM:RXNORM:2587892 | nirmatrelvir |
|  | cannot have |  | visit | UMLS:HL7V3.0:VisitType:IMP | Visit: Inpatient Encounter |
|  |  | or | procedure | UMLS:HCPCS:Q0220 | Injection, tixagevimab and cilgavimab, for the pre-exposure prophylaxis only, for certain adults and pediatric individuals (12 years of age and older weighing at least 40kg) with no known sars-cov-2 exposure, who either have moderate to severely compromised immune systems or for whom vaccination with any available covid-19 vaccine is not recommended due to a history of severe adverse reaction to a covid-19 vaccine(s) and/or covid-19 vaccine component(s), 300 mg |
|  |  | or | procedure | UMLS:HCPCS:M0220 | Injection, tixagevimab and cilgavimab, for the pre-exposure prophylaxis only, for certain adults and pediatric individuals (12 years of age and older weighing at least 40kg) with no known sars-cov-2 exposure, who either have moderate to severely compromised immune systems or for whom vaccination with any available covid-19 vaccine is not recommended due to a history of severe adverse reaction to a covid-19 vaccine(s) and/or covid-19 vaccine component(s), includes injection and post administration monitoring |
|  |  | or | procedure | UMLS:HCPCS:Q0222 | Injection, bebtelovimab, 175 mg |
|  |  | or | procedure | UMLS:HCPCS:M0222 | Intravenous injection, bebtelovimab, includes injection and post administration monitoring |
|  |  | or | procedure | UMLS:HCPCS:Q0239 | Injection, bamlanivimab-xxxx, 700 mg |
|  |  | or | procedure | UMLS:HCPCS:M0239 | Intravenous infusion, bamlanivimab-xxxx, includes infusion and post administration monitoring |

### Query Criteria for Cohort 2 (CVD, No NMV-r)

This query was run on the network Research with 80 HCO(s) queried and 80 HCO(s) responded. A total of 52 provider(s) responded with patients. The final cohort included 218,684 patients who matched the query criteria listed in the table below.

| Ungrouped terms | | | | | |
| --- | --- | --- | --- | --- | --- |
|  | must have |  | demographics | Age | Age (at least 18 years (most recent occurrence)) |
|  |  | and any of | medication | NLM:VA:CV100 | BETA BLOCKERS/RELATED |
|  |  |  | medication | NLM:VA:CV150 | ALPHA BLOCKERS/RELATED |
|  |  |  | medication | NLM:VA:CV200 | CALCIUM CHANNEL BLOCKERS |
|  |  |  | medication | NLM:VA:CV250 | ANTIANGINALS |
|  |  |  | medication | NLM:VA:CV350 | ANTILIPEMIC AGENTS |
|  |  |  | medication | NLM:VA:CV400 | ANTIHYPERTENSIVE COMBINATIONS |
|  |  |  | medication | NLM:VA:CV500 | PERIPHERAL VASODILATORS |
|  |  |  | medication | NLM:VA:CV700 | DIURETICS |
|  |  |  | medication | NLM:VA:CV800 | ACE INHIBITORS |
|  |  |  | medication | NLM:VA:CV805 | ANGIOTENSIN II INHIBITOR |
|  |  |  | diagnosis | UMLS:ICD10CM:I10-I16 | Hypertensive diseases |
|  |  |  | diagnosis | UMLS:ICD10CM:I20-I25 | Ischemic heart diseases |
|  |  |  | diagnosis | UMLS:ICD10CM:I26-I28 | Pulmonary heart disease and diseases of pulmonary circulation |
|  |  |  | diagnosis | UMLS:ICD10CM:I60-I69 | Cerebrovascular diseases |
|  |  |  | diagnosis | UMLS:ICD10CM:E11 | Type 2 diabetes mellitus |
|  |  |  | diagnosis | UMLS:ICD10CM:I42 | Cardiomyopathy |
|  |  |  | diagnosis | UMLS:ICD10CM:I43 | Cardiomyopathy in diseases classified elsewhere |
|  |  |  | diagnosis | UMLS:ICD10CM:I47 | Paroxysmal tachycardia |
|  |  |  | diagnosis | UMLS:ICD10CM:I48 | Atrial fibrillation and flutter |
|  |  |  | diagnosis | UMLS:ICD10CM:I49 | Other cardiac arrhythmias |
|  |  |  | diagnosis | UMLS:ICD10CM:I50 | Heart failure |
| Group 1 | | | | | |
|  | **Group 1A Vaccine** | | | | |
|  | must have | any of | procedure | UMLS:CPT:91300 | Severe acute respiratory syndrome coronavirus 2 (SARS-CoV-2) (Coronavirus disease [COVID-19]) vaccine, mRNA-LNP, spike protein, preservative free, 30 mcg/0.3mL dosage, diluent reconstituted, for intramuscular use |
|  |  |  | procedure | UMLS:CPT:91300 | Severe acute respiratory syndrome coronavirus 2 (SARS-CoV-2) (Coronavirus disease [COVID-19]) vaccine, mRNA-LNP, spike protein, preservative free, 30 mcg/0.3mL dosage, diluent reconstituted, for intramuscular use |
|  |  |  | procedure | UMLS:CPT:0001A | Immunization administration by intramuscular injection of severe acute respiratory syndrome coronavirus 2 (SARS-CoV-2) (Coronavirus disease [COVID-19]) vaccine, mRNA-LNP, spike protein, preservative free, 30 mcg/0.3mL dosage, diluent reconstituted; first dose |
|  |  |  | procedure | UMLS:CPT:0002A | Immunization administration by intramuscular injection of severe acute respiratory syndrome coronavirus 2 (SARS-CoV-2) (Coronavirus disease [COVID-19]) vaccine, mRNA-LNP, spike protein, preservative free, 30 mcg/0.3mL dosage, diluent reconstituted; second dose |
|  |  |  | procedure | UMLS:CPT:91301 | Severe acute respiratory syndrome coronavirus 2 (SARS-CoV-2) (Coronavirus disease [COVID-19]) vaccine, mRNA-LNP, spike protein, preservative free, 100 mcg/0.5mL dosage, for intramuscular use |
|  |  |  | procedure | UMLS:CPT:0011A | Immunization administration by intramuscular injection of severe acute respiratory syndrome coronavirus 2 (SARS-CoV-2) (Coronavirus disease [COVID-19]) vaccine, mRNA-LNP, spike protein, preservative free, 100 mcg/0.5mL dosage; first dose |
|  |  |  | procedure | UMLS:CPT:0012A | Immunization administration by intramuscular injection of severe acute respiratory syndrome coronavirus 2 (SARS-CoV-2) (Coronavirus disease [COVID-19]) vaccine, mRNA-LNP, spike protein, preservative free, 100 mcg/0.5mL dosage; second dose |
|  |  |  | procedure | UMLS:CPT:91303 | Severe acute respiratory syndrome coronavirus 2 (SARS-CoV-2) (coronavirus disease [COVID-19]) vaccine, DNA, spike protein, adenovirus type 26 (Ad26) vector, preservative free, 5x10^10 viral particles/0.5mL dosage, for intramuscular use |
|  |  |  | procedure | UMLS:CPT:0031A | Immunization administration by intramuscular injection of severe acute respiratory syndrome coronavirus 2 (SARS-CoV-2) (coronavirus disease [COVID-19]) vaccine, DNA, spike protein, adenovirus type 26 (Ad26) vector, preservative free, 5x10^10 viral particles/0.5mL dosage, single dose |
|  |  |  | procedure | UMLS:ICD10PCS:XW023S6 | Introduction of COVID-19 Vaccine Dose 1 into Muscle, Percutaneous Approach, New Technology Group 6 |
|  |  |  | procedure | UMLS:ICD10PCS:XW023T6 | Introduction of COVID-19 Vaccine Dose 2 into Muscle, Percutaneous Approach, New Technology Group 6 |
|  |  |  | procedure | UMLS:ICD10PCS:XW013S6 | Introduction of COVID-19 Vaccine Dose 1 into Subcutaneous Tissue, Percutaneous Approach, New Technology Group 6 |
|  |  |  | procedure | UMLS:ICD10PCS:XW013U6 | Introduction of COVID-19 Vaccine into Subcutaneous Tissue, Percutaneous Approach, New Technology Group 6 |
|  |  |  | procedure | UMLS:CPT:0021A | Immunization administration by intramuscular injection of severe acute respiratory syndrome coronavirus 2 (SARS-CoV-2) (coronavirus disease [COVID-19]) vaccine, DNA, spike protein, chimpanzee adenovirus Oxford 1 (ChAdOx1) vector, preservative free, 5x10^10 viral particles/0.5mL dosage; first dose |
|  |  |  | procedure | UMLS:CPT:0022A | Immunization administration by intramuscular injection of severe acute respiratory syndrome coronavirus 2 (SARS-CoV-2) (coronavirus disease [COVID-19]) vaccine, DNA, spike protein, chimpanzee adenovirus Oxford 1 (ChAdOx1) vector, preservative free, 5x10^10 viral particles/0.5mL dosage; second dose |
|  |  |  | procedure | UMLS:CPT:91302 | Severe acute respiratory syndrome coronavirus 2 (SARS-CoV-2) (coronavirus disease [COVID-19]) vaccine, DNA, spike protein, chimpanzee adenovirus Oxford 1 (ChAdOx1) vector, preservative free, 5x10^10 viral particles/0.5mL dosage, for intramuscular use |
|  |  |  | procedure | UMLS:HCPCS:U0003 | Infectious agent detection by nucleic acid (dna or rna); severe acute respiratory syndrome coronavirus 2 (sars-cov-2) (coronavirus disease [covid-19]), amplified probe technique, making use of high throughput technologies as described by cms-2020-01-r |
|  |  |  | medication | NLM:CVX:213 | SARS-CoV-2 (COVID-19) Vaccine |
|  |  |  | procedure | UMLS:CPT:0003A | Immunization administration by intramuscular injection of severe acute respiratory syndrome coronavirus 2 (SARS-CoV-2) (Coronavirus disease [COVID-19]) vaccine, mRNA-LNP, spike protein, preservative free, 30 mcg/0.3mL dosage, diluent reconstituted; third dose |
|  |  |  | medication | NLM:RXNORM:2479831 | SARS-COV-2 (COVID-19) vaccine, vector non-replicating |
|  |  |  | procedure | UMLS:CPT:0071A | Immunization administration by intramuscular injection of severe acute respiratory syndrome coronavirus 2 (SARS-CoV-2) (coronavirus disease [COVID-19]) vaccine, mRNA-LNP, spike protein, preservative free, 10 mcg/0.2 mL dosage, diluent reconstituted, tris-sucrose formulation; first dose |
|  |  |  | procedure | UMLS:CPT:91306 | Severe acute respiratory syndrome coronavirus 2 (SARS-CoV-2) (coronavirus disease [COVID-19]) vaccine, mRNA-LNP, spike protein, preservative free, 50 mcg/0.25 mL dosage, for intramuscular use |
|  |  |  | procedure | UMLS:CPT:0064A | Immunization administration by intramuscular injection of severe acute respiratory syndrome coronavirus 2 (SARS-CoV-2) (coronavirus disease [COVID-19]) vaccine, mRNA-LNP, spike protein, preservative free, 50 mcg/0.25 mL dosage, booster dose |
|  |  |  | procedure | UMLS:CPT:0072A | Immunization administration by intramuscular injection of severe acute respiratory syndrome coronavirus 2 (SARS-CoV-2) (coronavirus disease [COVID-19]) vaccine, mRNA-LNP, spike protein, preservative free, 10 mcg/0.2 mL dosage, diluent reconstituted, tris-sucrose formulation; second dose |
|  |  |  | procedure | UMLS:CPT:0013A | Immunization administration by intramuscular injection of severe acute respiratory syndrome coronavirus 2 (SARS-CoV-2) (coronavirus disease [COVID-19]) vaccine, mRNA-LNP, spike protein, preservative free, 100 mcg/0.5 mL dosage; third dose |
|  |  |  | procedure | UMLS:CPT:91305 | Severe acute respiratory syndrome coronavirus 2 (SARS-CoV-2) (coronavirus disease [COVID-19]) vaccine, mRNA-LNP, spike protein, preservative free, 30 mcg/0.3 mL dosage, tris-sucrose formulation, for intramuscular use |
|  |  |  | procedure | UMLS:ICD10PCS:XW023U6 | Introduction of COVID-19 Vaccine into Muscle, Percutaneous Approach, New Technology Group 6 |
|  |  |  | procedure | UMLS:CPT:0054A | Immunization administration by intramuscular injection of severe acute respiratory syndrome coronavirus 2 (SARS-CoV-2) (coronavirus disease [COVID-19]) vaccine, mRNA-LNP, spike protein, preservative free, 30 mcg/0.3 mL dosage, tris-sucrose formulation; booster dose |
|  |  |  | procedure | UMLS:CPT:0034A | Immunization administration by intramuscular injection of severe acute respiratory syndrome coronavirus 2 (SARS-CoV-2) (coronavirus disease [COVID-19]) vaccine, DNA, spike protein, adenovirus type 26 (Ad26) vector, preservative free, 5x10^10 viral particles/0.5 mL dosage; booster dose |
|  |  |  | procedure | UMLS:CPT:0051A | Immunization administration by intramuscular injection of severe acute respiratory syndrome coronavirus 2 (SARS-CoV-2) (coronavirus disease [COVID-19]) vaccine, mRNA-LNP, spike protein, preservative free, 30 mcg/0.3 mL dosage, tris-sucrose formulation; first dose |
|  |  |  | procedure | UMLS:CPT:0053A | Immunization administration by intramuscular injection of severe acute respiratory syndrome coronavirus 2 (SARS-CoV-2) (coronavirus disease [COVID-19]) vaccine, mRNA-LNP, spike protein, preservative free, 30 mcg/0.3 mL dosage, tris-sucrose formulation; third dose |
|  |  |  | procedure | UMLS:CPT:0052A | Immunization administration by intramuscular injection of severe acute respiratory syndrome coronavirus 2 (SARS-CoV-2) (coronavirus disease [COVID-19]) vaccine, mRNA-LNP, spike protein, preservative free, 30 mcg/0.3 mL dosage, tris-sucrose formulation; second dose |
|  |  |  | procedure | UMLS:ICD10PCS:XW013T6 | Introduction of COVID-19 Vaccine Dose 2 into Subcutaneous Tissue, Percutaneous Approach, New Technology Group 6 |
|  | date constraint | | The terms in this group occurred on or before Dec 31, 2022 | | |
|  | event relationship | | Any instance of Group 1B occurred at least 1 month after any instance of Vaccine | | |
|  | **Group 1B** | | | | |
|  | must have | any of | laboratory | TNX:9088 | SARS coronavirus 2 and related RNA [Presence] (labResult: Positive) |
|  |  |  | laboratory | UMLS:LNC:94500-6 | SARS-CoV-2 (COVID19) RNA [Presence] in Respiratory specimen by NAA with probe detection (labResult: Positive) |
|  |  |  | laboratory | TNX:9089 | SARS coronavirus 2 IgG IgM Ab [Presence] in Serum or Plasma (labResult: Positive) |
|  |  |  | laboratory | TNX:9088 | SARS coronavirus 2 and related RNA [Presence] (labResult: Positive) |
|  |  |  | diagnosis | UMLS:ICD10CM:U07.1 | COVID-19 |
|  |  |  | laboratory | TNX:9088 | SARS coronavirus 2 and related RNA [Presence] (labResult: Positive) |
|  |  |  | laboratory | UMLS:LNC:94500-6 | SARS-CoV-2 (COVID19) RNA [Presence] in Respiratory specimen by NAA with probe detection (labResult: Positive) |
|  |  |  | laboratory | UMLS:LNC:94309-2 | SARS-CoV-2 (COVID-19) RNA [Presence] in Specimen by NAA with probe detection (labResult: Positive) |
|  |  |  | laboratory | UMLS:LNC:94534-5 | SARS-CoV-2 (COVID-19) RdRp gene [Presence] in Respiratory specimen by NAA with probe detection (labResult: Positive) |
|  |  |  | laboratory | UMLS:LNC:94565-9 | SARS-CoV-2 (COVID-19) RNA [Presence] in Nasopharynx by NAA with non-probe detection (labResult: Positive) |
|  |  |  | laboratory | UMLS:LNC:94316-7 | SARS-CoV-2 (COVID-19) N gene [Presence] in Specimen by NAA with probe detection (labResult: Positive) |
|  |  |  | laboratory | UMLS:LNC:95209-3 | SARS-CoV+SARS-CoV-2 (COVID-19) Ag [Presence] in Respiratory specimen by Rapid immunoassay (labResult: Positive) |
|  |  |  | laboratory | UMLS:LNC:94559-2 | SARS-CoV-2 (COVID-19) ORF1ab region [Presence] in Respiratory specimen by NAA with probe detection (labResult: Positive) |
|  |  |  | laboratory | UMLS:LNC:94759-8 | SARS-CoV-2 (COVID-19) RNA [Presence] in Nasopharynx by NAA with probe detection (labResult: Positive) |
|  |  |  | laboratory | UMLS:LNC:94558-4 | SARS-CoV-2 (COVID-19) Ag [Presence] in Respiratory specimen by Rapid immunoassay (labResult: Positive) |
|  |  |  | laboratory | UMLS:LNC:95608-6 | SARS-CoV-2 (COVID-19) RNA [Presence] in Respiratory specimen by NAA with non-probe detection (labResult: Positive) |
|  |  |  | laboratory | UMLS:LNC:94508-9 | SARS-CoV-2 (COVID-19) IgM Ab [Presence] in Serum, Plasma or Blood by Rapid immunoassay (labResult: Positive) |
|  |  |  | laboratory | UMLS:LNC:94763-0 | SARS-CoV-2 (COVID-19) [Presence] in Specimen by Organism specific culture (labResult: Positive) |
|  |  |  | laboratory | UMLS:LNC:96119-3 | SARS-CoV-2 (COVID-19) Ag [Presence] in Upper respiratory specimen by Immunoassay (labResult: Positive) |
|  |  |  | laboratory | UMLS:LNC:94533-7 | SARS-CoV-2 (COVID-19) N gene [Presence] in Respiratory specimen by NAA with probe detection (labResult: Positive) |
|  |  |  | laboratory | UMLS:LNC:94564-2 | SARS-CoV-2 (COVID-19) IgM Ab [Presence] in Serum or Plasma by Immunoassay (labResult: Positive) |
|  |  |  | laboratory | UMLS:LNC:95406-5 | SARS-CoV-2 (COVID-19) RNA [Presence] in Nose by NAA with probe detection (labResult: Positive) |
|  |  |  | laboratory | UMLS:LNC:94758-0 | SARS-related coronavirus E gene [Presence] in Respiratory specimen by NAA with probe detection (labResult: Positive) |
|  |  |  | laboratory | UMLS:LNC:96763-8 | SARS-CoV-2 (COVID-19) E gene [Presence] in Respiratory specimen by NAA with probe detection (labResult: Positive) |
|  |  |  | laboratory | UMLS:LNC:94502-2 | SARS-related coronavirus RNA [Presence] in Respiratory specimen by NAA with probe detection (labResult: Positive) |
|  |  |  | laboratory | UMLS:LNC:94760-6 | SARS-CoV-2 (COVID-19) N gene [Presence] in Nasopharynx by NAA with probe detection (labResult: Positive) |
|  |  |  | laboratory | UMLS:LNC:94314-2 | SARS-CoV-2 (COVID-19) RdRp gene [Presence] in Specimen by NAA with probe detection (labResult: Positive) |
|  |  |  | laboratory | UMLS:LNC:97097-0 | SARS-CoV-2 (COVID-19) Ag [Presence] in Upper respiratory specimen by Rapid immunoassay (labResult: Positive) |
|  |  |  | laboratory | UMLS:LNC:94647-5 | SARS-related coronavirus RNA [Presence] in Specimen by NAA with probe detection (labResult: Positive) |
|  |  |  | laboratory | UMLS:LNC:94845-5 | SARS-CoV-2 (COVID-19) RNA [Presence] in Saliva (oral fluid) by NAA with probe detection (labResult: Positive) |
|  |  |  | laboratory | UMLS:LNC:95409-9 | SARS-CoV-2 (COVID-19) N gene [Presence] in Nose by NAA with probe detection (labResult: Positive) |
|  |  |  | diagnosis | UMLS:ICD10CM:U07.1 | COVID-19 |
| Group 2 | | | | | |
|  | **Group 2A** | | | | |
|  | must have | any of | laboratory | TNX:9088 | SARS coronavirus 2 and related RNA [Presence] (labResult: Positive) |
|  |  |  | laboratory | UMLS:LNC:94500-6 | SARS-CoV-2 (COVID19) RNA [Presence] in Respiratory specimen by NAA with probe detection (labResult: Positive) |
|  |  |  | laboratory | TNX:9089 | SARS coronavirus 2 IgG IgM Ab [Presence] in Serum or Plasma (labResult: Positive) |
|  |  |  | laboratory | TNX:9088 | SARS coronavirus 2 and related RNA [Presence] (labResult: Positive) |
|  |  |  | diagnosis | UMLS:ICD10CM:U07.1 | COVID-19 |
|  |  |  | laboratory | TNX:9088 | SARS coronavirus 2 and related RNA [Presence] (labResult: Positive) |
|  |  |  | laboratory | UMLS:LNC:94500-6 | SARS-CoV-2 (COVID19) RNA [Presence] in Respiratory specimen by NAA with probe detection (labResult: Positive) |
|  |  |  | laboratory | UMLS:LNC:94309-2 | SARS-CoV-2 (COVID-19) RNA [Presence] in Specimen by NAA with probe detection (labResult: Positive) |
|  |  |  | laboratory | UMLS:LNC:94534-5 | SARS-CoV-2 (COVID-19) RdRp gene [Presence] in Respiratory specimen by NAA with probe detection (labResult: Positive) |
|  |  |  | laboratory | UMLS:LNC:94565-9 | SARS-CoV-2 (COVID-19) RNA [Presence] in Nasopharynx by NAA with non-probe detection (labResult: Positive) |
|  |  |  | laboratory | UMLS:LNC:94316-7 | SARS-CoV-2 (COVID-19) N gene [Presence] in Specimen by NAA with probe detection (labResult: Positive) |
|  |  |  | laboratory | UMLS:LNC:95209-3 | SARS-CoV+SARS-CoV-2 (COVID-19) Ag [Presence] in Respiratory specimen by Rapid immunoassay (labResult: Positive) |
|  |  |  | laboratory | UMLS:LNC:94559-2 | SARS-CoV-2 (COVID-19) ORF1ab region [Presence] in Respiratory specimen by NAA with probe detection (labResult: Positive) |
|  |  |  | laboratory | UMLS:LNC:94759-8 | SARS-CoV-2 (COVID-19) RNA [Presence] in Nasopharynx by NAA with probe detection (labResult: Positive) |
|  |  |  | laboratory | UMLS:LNC:94558-4 | SARS-CoV-2 (COVID-19) Ag [Presence] in Respiratory specimen by Rapid immunoassay (labResult: Positive) |
|  |  |  | laboratory | UMLS:LNC:95608-6 | SARS-CoV-2 (COVID-19) RNA [Presence] in Respiratory specimen by NAA with non-probe detection (labResult: Positive) |
|  |  |  | laboratory | UMLS:LNC:94508-9 | SARS-CoV-2 (COVID-19) IgM Ab [Presence] in Serum, Plasma or Blood by Rapid immunoassay (labResult: Positive) |
|  |  |  | laboratory | UMLS:LNC:94763-0 | SARS-CoV-2 (COVID-19) [Presence] in Specimen by Organism specific culture (labResult: Positive) |
|  |  |  | laboratory | UMLS:LNC:96119-3 | SARS-CoV-2 (COVID-19) Ag [Presence] in Upper respiratory specimen by Immunoassay (labResult: Positive) |
|  |  |  | laboratory | UMLS:LNC:94533-7 | SARS-CoV-2 (COVID-19) N gene [Presence] in Respiratory specimen by NAA with probe detection (labResult: Positive) |
|  |  |  | laboratory | UMLS:LNC:94564-2 | SARS-CoV-2 (COVID-19) IgM Ab [Presence] in Serum or Plasma by Immunoassay (labResult: Positive) |
|  |  |  | laboratory | UMLS:LNC:95406-5 | SARS-CoV-2 (COVID-19) RNA [Presence] in Nose by NAA with probe detection (labResult: Positive) |
|  |  |  | laboratory | UMLS:LNC:94758-0 | SARS-related coronavirus E gene [Presence] in Respiratory specimen by NAA with probe detection (labResult: Positive) |
|  |  |  | laboratory | UMLS:LNC:96763-8 | SARS-CoV-2 (COVID-19) E gene [Presence] in Respiratory specimen by NAA with probe detection (labResult: Positive) |
|  |  |  | laboratory | UMLS:LNC:94502-2 | SARS-related coronavirus RNA [Presence] in Respiratory specimen by NAA with probe detection (labResult: Positive) |
|  |  |  | laboratory | UMLS:LNC:94760-6 | SARS-CoV-2 (COVID-19) N gene [Presence] in Nasopharynx by NAA with probe detection (labResult: Positive) |
|  |  |  | laboratory | UMLS:LNC:94314-2 | SARS-CoV-2 (COVID-19) RdRp gene [Presence] in Specimen by NAA with probe detection (labResult: Positive) |
|  |  |  | laboratory | UMLS:LNC:97097-0 | SARS-CoV-2 (COVID-19) Ag [Presence] in Upper respiratory specimen by Rapid immunoassay (labResult: Positive) |
|  |  |  | laboratory | UMLS:LNC:94647-5 | SARS-related coronavirus RNA [Presence] in Specimen by NAA with probe detection (labResult: Positive) |
|  |  |  | laboratory | UMLS:LNC:94845-5 | SARS-CoV-2 (COVID-19) RNA [Presence] in Saliva (oral fluid) by NAA with probe detection (labResult: Positive) |
|  |  |  | laboratory | UMLS:LNC:95409-9 | SARS-CoV-2 (COVID-19) N gene [Presence] in Nose by NAA with probe detection (labResult: Positive) |
|  |  |  | diagnosis | UMLS:ICD10CM:U07.1 | COVID-19 |
|  | date constraint | | The terms in this group occurred at any time | | |
|  | event relationship | | Any instance of Group 2B occurred within 5 days on or after any instance of Group 2A | | |
|  | **Group 2B** | | | | |
|  | cannot have |  | medication | NLM:RXNORM:85762 | ritonavir |
|  |  | or | medication | NLM:RXNORM:2587892 | nirmatrelvir |
|  |  | or | visit | UMLS:HL7V3.0:VisitType:IMP | Visit: Inpatient Encounter |
|  |  | or | procedure | UMLS:HCPCS:Q0220 | Injection, tixagevimab and cilgavimab, for the pre-exposure prophylaxis only, for certain adults and pediatric individuals (12 years of age and older weighing at least 40kg) with no known sars-cov-2 exposure, who either have moderate to severely compromised immune systems or for whom vaccination with any available covid-19 vaccine is not recommended due to a history of severe adverse reaction to a covid-19 vaccine(s) and/or covid-19 vaccine component(s), 300 mg |
|  |  | or | procedure | UMLS:HCPCS:M0220 | Injection, tixagevimab and cilgavimab, for the pre-exposure prophylaxis only, for certain adults and pediatric individuals (12 years of age and older weighing at least 40kg) with no known sars-cov-2 exposure, who either have moderate to severely compromised immune systems or for whom vaccination with any available covid-19 vaccine is not recommended due to a history of severe adverse reaction to a covid-19 vaccine(s) and/or covid-19 vaccine component(s), includes injection and post administration monitoring |
|  |  | or | procedure | UMLS:HCPCS:Q0222 | Injection, bebtelovimab, 175 mg |
|  |  | or | procedure | UMLS:HCPCS:M0222 | Intravenous injection, bebtelovimab, includes injection and post administration monitoring |
|  |  | or | procedure | UMLS:HCPCS:Q0239 | Injection, bamlanivimab-xxxx, 700 mg |
|  |  | or | procedure | UMLS:HCPCS:M0239 | Intravenous infusion, bamlanivimab-xxxx, includes infusion and post administration monitoring |

## Analysis Setup

This section contains the Index Event and Time Window definitions and a list of selected outcomes and the analyses.

### Index Event & Time Window Definitions

The index event defines the point in time when each patient in the cohort enters the analysis. To define an index event for the cohort, one or more criteria for the cohort must be selected. The index date for each patient within a cohort is the day on which the patient first met the selected criteria for the cohort (listed in the table below).

As the index event defines the earliest time point after which outcomes are analyzed, the time window defines the duration during which outcomes are analyzed. The time window can start on the same day as the index event or at any specified time interval after the index event. The time window can end any time after the start date. Outcomes are defined as diagnoses, medications, procedures, or laboratory values that happened in the time window starting after the first occurrence of the index event.

### Index Events Used in this Analysis

Index events for the Compare Outcomes analysis were derived from the cohort definitions. Index events were defined separately for each cohort and were based on the criteria used in the original cohort definition. Please see Appendix B for the text representation of the index event definition.

The index event for Cohort 1 (query name: CVD, Yes Pax, Dec 31 2022) was defined as the following:

|  | | | | | |
| --- | --- | --- | --- | --- | --- |
| Group 1 | | | | | |
|  | **Group 1A Group 2A** | | | | |
|  | must have | any of | laboratory | TNX:9088 | SARS coronavirus 2 and related RNA [Presence] (labResult: Positive) |
|  |  |  | laboratory | UMLS:LNC:94500-6 | SARS-CoV-2 (COVID19) RNA [Presence] in Respiratory specimen by NAA with probe detection (labResult: Positive) |
|  |  |  | laboratory | TNX:9089 | SARS coronavirus 2 IgG IgM Ab [Presence] in Serum or Plasma (labResult: Positive) |
|  |  |  | laboratory | TNX:9088 | SARS coronavirus 2 and related RNA [Presence] (labResult: Positive) |
|  |  |  | diagnosis | UMLS:ICD10CM:U07.1 | COVID-19 |
|  |  |  | laboratory | TNX:9088 | SARS coronavirus 2 and related RNA [Presence] (labResult: Positive) |
|  |  |  | laboratory | UMLS:LNC:94500-6 | SARS-CoV-2 (COVID19) RNA [Presence] in Respiratory specimen by NAA with probe detection (labResult: Positive) |
|  |  |  | laboratory | UMLS:LNC:94309-2 | SARS-CoV-2 (COVID-19) RNA [Presence] in Specimen by NAA with probe detection (labResult: Positive) |
|  |  |  | laboratory | UMLS:LNC:94534-5 | SARS-CoV-2 (COVID-19) RdRp gene [Presence] in Respiratory specimen by NAA with probe detection (labResult: Positive) |
|  |  |  | laboratory | UMLS:LNC:94565-9 | SARS-CoV-2 (COVID-19) RNA [Presence] in Nasopharynx by NAA with non-probe detection (labResult: Positive) |
|  |  |  | laboratory | UMLS:LNC:94316-7 | SARS-CoV-2 (COVID-19) N gene [Presence] in Specimen by NAA with probe detection (labResult: Positive) |
|  |  |  | laboratory | UMLS:LNC:95209-3 | SARS-CoV+SARS-CoV-2 (COVID-19) Ag [Presence] in Respiratory specimen by Rapid immunoassay (labResult: Positive) |
|  |  |  | laboratory | UMLS:LNC:94559-2 | SARS-CoV-2 (COVID-19) ORF1ab region [Presence] in Respiratory specimen by NAA with probe detection (labResult: Positive) |
|  |  |  | laboratory | UMLS:LNC:94759-8 | SARS-CoV-2 (COVID-19) RNA [Presence] in Nasopharynx by NAA with probe detection (labResult: Positive) |
|  |  |  | laboratory | UMLS:LNC:94558-4 | SARS-CoV-2 (COVID-19) Ag [Presence] in Respiratory specimen by Rapid immunoassay (labResult: Positive) |
|  |  |  | laboratory | UMLS:LNC:95608-6 | SARS-CoV-2 (COVID-19) RNA [Presence] in Respiratory specimen by NAA with non-probe detection (labResult: Positive) |
|  |  |  | laboratory | UMLS:LNC:94508-9 | SARS-CoV-2 (COVID-19) IgM Ab [Presence] in Serum, Plasma or Blood by Rapid immunoassay (labResult: Positive) |
|  |  |  | laboratory | UMLS:LNC:94763-0 | SARS-CoV-2 (COVID-19) [Presence] in Specimen by Organism specific culture (labResult: Positive) |
|  |  |  | laboratory | UMLS:LNC:96119-3 | SARS-CoV-2 (COVID-19) Ag [Presence] in Upper respiratory specimen by Immunoassay (labResult: Positive) |
|  |  |  | laboratory | UMLS:LNC:94533-7 | SARS-CoV-2 (COVID-19) N gene [Presence] in Respiratory specimen by NAA with probe detection (labResult: Positive) |
|  |  |  | laboratory | UMLS:LNC:94564-2 | SARS-CoV-2 (COVID-19) IgM Ab [Presence] in Serum or Plasma by Immunoassay (labResult: Positive) |
|  |  |  | laboratory | UMLS:LNC:95406-5 | SARS-CoV-2 (COVID-19) RNA [Presence] in Nose by NAA with probe detection (labResult: Positive) |
|  |  |  | laboratory | UMLS:LNC:94758-0 | SARS-related coronavirus E gene [Presence] in Respiratory specimen by NAA with probe detection (labResult: Positive) |
|  |  |  | laboratory | UMLS:LNC:96763-8 | SARS-CoV-2 (COVID-19) E gene [Presence] in Respiratory specimen by NAA with probe detection (labResult: Positive) |
|  |  |  | laboratory | UMLS:LNC:94502-2 | SARS-related coronavirus RNA [Presence] in Respiratory specimen by NAA with probe detection (labResult: Positive) |
|  |  |  | laboratory | UMLS:LNC:94760-6 | SARS-CoV-2 (COVID-19) N gene [Presence] in Nasopharynx by NAA with probe detection (labResult: Positive) |
|  |  |  | laboratory | UMLS:LNC:94314-2 | SARS-CoV-2 (COVID-19) RdRp gene [Presence] in Specimen by NAA with probe detection (labResult: Positive) |
|  |  |  | laboratory | UMLS:LNC:97097-0 | SARS-CoV-2 (COVID-19) Ag [Presence] in Upper respiratory specimen by Rapid immunoassay (labResult: Positive) |
|  |  |  | laboratory | UMLS:LNC:94647-5 | SARS-related coronavirus RNA [Presence] in Specimen by NAA with probe detection (labResult: Positive) |
|  |  |  | laboratory | UMLS:LNC:94845-5 | SARS-CoV-2 (COVID-19) RNA [Presence] in Saliva (oral fluid) by NAA with probe detection (labResult: Positive) |
|  |  |  | laboratory | UMLS:LNC:95409-9 | SARS-CoV-2 (COVID-19) N gene [Presence] in Nose by NAA with probe detection (labResult: Positive) |
|  |  |  | diagnosis | UMLS:ICD10CM:U07.1 | COVID-19 |
|  | date constraint | | The terms in this group occurred at any time | | |
|  | event relationship | | Any instance of Group 2B occurred within 5 days on or after any instance of Group 2A | | |
|  | **Group 1B Group 2B** | | | | |
|  | must have |  | medication | NLM:RXNORM:85762 | ritonavir |
|  |  | and | medication | NLM:RXNORM:2587892 | nirmatrelvir |
|  | cannot have |  | visit | UMLS:HL7V3.0:VisitType:IMP | Visit: Inpatient Encounter |
|  |  | or | procedure | UMLS:HCPCS:Q0220 | Injection, tixagevimab and cilgavimab, for the pre-exposure prophylaxis only, for certain adults and pediatric individuals (12 years of age and older weighing at least 40kg) with no known sars-cov-2 exposure, who either have moderate to severely compromised immune systems or for whom vaccination with any available covid-19 vaccine is not recommended due to a history of severe adverse reaction to a covid-19 vaccine(s) and/or covid-19 vaccine component(s), 300 mg |
|  |  | or | procedure | UMLS:HCPCS:M0220 | Injection, tixagevimab and cilgavimab, for the pre-exposure prophylaxis only, for certain adults and pediatric individuals (12 years of age and older weighing at least 40kg) with no known sars-cov-2 exposure, who either have moderate to severely compromised immune systems or for whom vaccination with any available covid-19 vaccine is not recommended due to a history of severe adverse reaction to a covid-19 vaccine(s) and/or covid-19 vaccine component(s), includes injection and post administration monitoring |
|  |  | or | procedure | UMLS:HCPCS:Q0222 | Injection, bebtelovimab, 175 mg |
|  |  | or | procedure | UMLS:HCPCS:M0222 | Intravenous injection, bebtelovimab, includes injection and post administration monitoring |
|  |  | or | procedure | UMLS:HCPCS:Q0239 | Injection, bamlanivimab-xxxx, 700 mg |
|  |  | or | procedure | UMLS:HCPCS:M0239 | Intravenous infusion, bamlanivimab-xxxx, includes infusion and post administration monitoring |

The index event for Cohort 2 was defined as the following:

|  | | | | | |
| --- | --- | --- | --- | --- | --- |
| Group 1 | | | | | |
|  | **Group 1A Group 2A** | | | | |
|  | must have | any of | laboratory | TNX:9088 | SARS coronavirus 2 and related RNA [Presence] (labResult: Positive) |
|  |  |  | laboratory | UMLS:LNC:94500-6 | SARS-CoV-2 (COVID19) RNA [Presence] in Respiratory specimen by NAA with probe detection (labResult: Positive) |
|  |  |  | laboratory | TNX:9089 | SARS coronavirus 2 IgG IgM Ab [Presence] in Serum or Plasma (labResult: Positive) |
|  |  |  | laboratory | TNX:9088 | SARS coronavirus 2 and related RNA [Presence] (labResult: Positive) |
|  |  |  | diagnosis | UMLS:ICD10CM:U07.1 | COVID-19 |
|  |  |  | laboratory | TNX:9088 | SARS coronavirus 2 and related RNA [Presence] (labResult: Positive) |
|  |  |  | laboratory | UMLS:LNC:94500-6 | SARS-CoV-2 (COVID19) RNA [Presence] in Respiratory specimen by NAA with probe detection (labResult: Positive) |
|  |  |  | laboratory | UMLS:LNC:94309-2 | SARS-CoV-2 (COVID-19) RNA [Presence] in Specimen by NAA with probe detection (labResult: Positive) |
|  |  |  | laboratory | UMLS:LNC:94534-5 | SARS-CoV-2 (COVID-19) RdRp gene [Presence] in Respiratory specimen by NAA with probe detection (labResult: Positive) |
|  |  |  | laboratory | UMLS:LNC:94565-9 | SARS-CoV-2 (COVID-19) RNA [Presence] in Nasopharynx by NAA with non-probe detection (labResult: Positive) |
|  |  |  | laboratory | UMLS:LNC:94316-7 | SARS-CoV-2 (COVID-19) N gene [Presence] in Specimen by NAA with probe detection (labResult: Positive) |
|  |  |  | laboratory | UMLS:LNC:95209-3 | SARS-CoV+SARS-CoV-2 (COVID-19) Ag [Presence] in Respiratory specimen by Rapid immunoassay (labResult: Positive) |
|  |  |  | laboratory | UMLS:LNC:94559-2 | SARS-CoV-2 (COVID-19) ORF1ab region [Presence] in Respiratory specimen by NAA with probe detection (labResult: Positive) |
|  |  |  | laboratory | UMLS:LNC:94759-8 | SARS-CoV-2 (COVID-19) RNA [Presence] in Nasopharynx by NAA with probe detection (labResult: Positive) |
|  |  |  | laboratory | UMLS:LNC:94558-4 | SARS-CoV-2 (COVID-19) Ag [Presence] in Respiratory specimen by Rapid immunoassay (labResult: Positive) |
|  |  |  | laboratory | UMLS:LNC:95608-6 | SARS-CoV-2 (COVID-19) RNA [Presence] in Respiratory specimen by NAA with non-probe detection (labResult: Positive) |
|  |  |  | laboratory | UMLS:LNC:94508-9 | SARS-CoV-2 (COVID-19) IgM Ab [Presence] in Serum, Plasma or Blood by Rapid immunoassay (labResult: Positive) |
|  |  |  | laboratory | UMLS:LNC:94763-0 | SARS-CoV-2 (COVID-19) [Presence] in Specimen by Organism specific culture (labResult: Positive) |
|  |  |  | laboratory | UMLS:LNC:96119-3 | SARS-CoV-2 (COVID-19) Ag [Presence] in Upper respiratory specimen by Immunoassay (labResult: Positive) |
|  |  |  | laboratory | UMLS:LNC:94533-7 | SARS-CoV-2 (COVID-19) N gene [Presence] in Respiratory specimen by NAA with probe detection (labResult: Positive) |
|  |  |  | laboratory | UMLS:LNC:94564-2 | SARS-CoV-2 (COVID-19) IgM Ab [Presence] in Serum or Plasma by Immunoassay (labResult: Positive) |
|  |  |  | laboratory | UMLS:LNC:95406-5 | SARS-CoV-2 (COVID-19) RNA [Presence] in Nose by NAA with probe detection (labResult: Positive) |
|  |  |  | laboratory | UMLS:LNC:94758-0 | SARS-related coronavirus E gene [Presence] in Respiratory specimen by NAA with probe detection (labResult: Positive) |
|  |  |  | laboratory | UMLS:LNC:96763-8 | SARS-CoV-2 (COVID-19) E gene [Presence] in Respiratory specimen by NAA with probe detection (labResult: Positive) |
|  |  |  | laboratory | UMLS:LNC:94502-2 | SARS-related coronavirus RNA [Presence] in Respiratory specimen by NAA with probe detection (labResult: Positive) |
|  |  |  | laboratory | UMLS:LNC:94760-6 | SARS-CoV-2 (COVID-19) N gene [Presence] in Nasopharynx by NAA with probe detection (labResult: Positive) |
|  |  |  | laboratory | UMLS:LNC:94314-2 | SARS-CoV-2 (COVID-19) RdRp gene [Presence] in Specimen by NAA with probe detection (labResult: Positive) |
|  |  |  | laboratory | UMLS:LNC:97097-0 | SARS-CoV-2 (COVID-19) Ag [Presence] in Upper respiratory specimen by Rapid immunoassay (labResult: Positive) |
|  |  |  | laboratory | UMLS:LNC:94647-5 | SARS-related coronavirus RNA [Presence] in Specimen by NAA with probe detection (labResult: Positive) |
|  |  |  | laboratory | UMLS:LNC:94845-5 | SARS-CoV-2 (COVID-19) RNA [Presence] in Saliva (oral fluid) by NAA with probe detection (labResult: Positive) |
|  |  |  | laboratory | UMLS:LNC:95409-9 | SARS-CoV-2 (COVID-19) N gene [Presence] in Nose by NAA with probe detection (labResult: Positive) |
|  |  |  | diagnosis | UMLS:ICD10CM:U07.1 | COVID-19 |
|  | date constraint | | The terms in this group occurred at any time | | |
|  | event relationship | | Any instance of Group 2B occurred within 5 days on or after any instance of Group 2A | | |
|  | **Group 1B Group 2B** | | | | |
|  | cannot have |  | medication | NLM:RXNORM:85762 | ritonavir |
|  |  | or | medication | NLM:RXNORM:2587892 | nirmatrelvir |
|  |  | or | visit | UMLS:HL7V3.0:VisitType:IMP | Visit: Inpatient Encounter |
|  |  | or | procedure | UMLS:HCPCS:Q0220 | Injection, tixagevimab and cilgavimab, for the pre-exposure prophylaxis only, for certain adults and pediatric individuals (12 years of age and older weighing at least 40kg) with no known sars-cov-2 exposure, who either have moderate to severely compromised immune systems or for whom vaccination with any available covid-19 vaccine is not recommended due to a history of severe adverse reaction to a covid-19 vaccine(s) and/or covid-19 vaccine component(s), 300 mg |
|  |  | or | procedure | UMLS:HCPCS:M0220 | Injection, tixagevimab and cilgavimab, for the pre-exposure prophylaxis only, for certain adults and pediatric individuals (12 years of age and older weighing at least 40kg) with no known sars-cov-2 exposure, who either have moderate to severely compromised immune systems or for whom vaccination with any available covid-19 vaccine is not recommended due to a history of severe adverse reaction to a covid-19 vaccine(s) and/or covid-19 vaccine component(s), includes injection and post administration monitoring |
|  |  | or | procedure | UMLS:HCPCS:Q0222 | Injection, bebtelovimab, 175 mg |
|  |  | or | procedure | UMLS:HCPCS:M0222 | Intravenous injection, bebtelovimab, includes injection and post administration monitoring |
|  |  | or | procedure | UMLS:HCPCS:Q0239 | Injection, bamlanivimab-xxxx, 700 mg |
|  |  | or | procedure | UMLS:HCPCS:M0239 | Intravenous infusion, bamlanivimab-xxxx, includes infusion and post administration monitoring |

### Outcome Definitions

Table below outlines the definitions for each outcome and the analysis specifications. For outcome definitions consisting of more than one term, at least one term must match.

| Broadly defined PASC | | | | |
| --- | --- | --- | --- | --- |
|  | **Outcome definition** | | | |
|  | | Diagnosis | UMLS:ICD10CM:F40-F48 | Anxiety, dissociative, stress-related, somatoform and other nonpsychotic mental disorders |
|  | | Diagnosis | UMLS:ICD10CM:F50-F59 | Behavioral syndromes associated with physiological disturbances and physical factors |
|  | | Diagnosis | UMLS:ICD10CM:R00-R09 | Symptoms and signs involving the circulatory and respiratory systems |
|  | | Diagnosis | UMLS:ICD10CM:R10-R19 | Symptoms and signs involving the digestive system and abdomen |
|  | | Diagnosis | UMLS:ICD10CM:R25-R29 | Symptoms and signs involving the nervous and musculoskeletal systems |
|  | | Diagnosis | UMLS:ICD10CM:R40-R46 | Symptoms and signs involving cognition, perception, emotional state and behavior |
|  | | Diagnosis | UMLS:ICD10CM:R50 | Fever of other and unknown origin |
|  | | Diagnosis | UMLS:ICD10CM:R51 | Headache |
|  | | Diagnosis | UMLS:ICD10CM:R52 | Pain, unspecified |
|  | | Diagnosis | UMLS:ICD10CM:R53 | Malaise and fatigue |
|  | | Diagnosis | UMLS:ICD10CM:R63 | Symptoms and signs concerning food and fluid intake |
|  | | Diagnosis | UMLS:ICD10CM:G93.3 | Postviral fatigue syndrome |

| Narrowly defined PASC – 3 symptoms cluster | | | | |
| --- | --- | --- | --- | --- |
|  | **Outcome definition** | | | |
|  | | Diagnosis | UMLS:ICD10CM:R05 | Cough |
|  | | Diagnosis | UMLS:ICD10CM:R06 | Abnormalities of breathing |
|  | | Diagnosis | UMLS:ICD10CM:R07.0 | Pain in throat |
|  | | Diagnosis | UMLS:ICD10CM:R07.1 | Chest pain on breathing |
|  | | Diagnosis | UMLS:ICD10CM:R09.1 | Pleurisy |
|  | | Diagnosis | UMLS:ICD10CM:R09.2 | Respiratory arrest |
|  | | Diagnosis | UMLS:ICD10CM:R09.81 | Nasal congestion |
|  | | Diagnosis | UMLS:ICD10CM:R09.82 | Postnasal drip |
|  | | Diagnosis | UMLS:ICD10CM:R40 | Somnolence, stupor and coma |
|  | | Diagnosis | UMLS:ICD10CM:R41 | Other symptoms and signs involving cognitive functions and awareness |
|  | | Diagnosis | UMLS:ICD10CM:R52 | Pain, unspecified |
|  | | Diagnosis | UMLS:ICD10CM:R53.1 | Weakness |
|  | | Diagnosis | UMLS:ICD10CM:R53.8 | Other malaise and fatigue |
|  | | Diagnosis | UMLS:ICD10CM:M79.1 | Myalgia |
|  | | Diagnosis | UMLS:ICD10CM:F30-F39 | Mood [affective] disorders |
|  | | Diagnosis | UMLS:ICD10CM:F40-F48 | Anxiety, dissociative, stress-related, somatoform and other nonpsychotic mental disorders |
|  | | Diagnosis | UMLS:ICD10CM:F50-F59 | Behavioral syndromes associated with physiological disturbances and physical factors |

| Constitutional symptoms - fatigue/malaise/headache/fever/weakness/poor-fluid intake | | | | |
| --- | --- | --- | --- | --- |
|  | **Outcome definition** | | | |
|  | | Diagnosis | UMLS:ICD10CM:R50 | Fever of other and unknown origin |
|  | | Diagnosis | UMLS:ICD10CM:R51 | Headache |
|  | | Diagnosis | UMLS:ICD10CM:R52 | Pain, unspecified |
|  | | Diagnosis | UMLS:ICD10CM:R53 | Malaise and fatigue |
|  | | Diagnosis | UMLS:ICD10CM:R63 | Symptoms and signs concerning food and fluid intake |
|  | | Diagnosis | UMLS:ICD10CM:R53 | Malaise and fatigue |
|  | | Diagnosis | UMLS:ICD10CM:R53.83 | Other fatigue |
|  | | Diagnosis | UMLS:ICD10CM:G93.3 | Postviral fatigue syndrome |
|  | | Diagnosis | UMLS:ICD10CM:R53.1 | Weakness |
|  |  |  |  |  |
| CV and respiratory symptoms | | | | |
|  | **Outcome definition** | | | |
|  | | Diagnosis | UMLS:ICD10CM:R00-R09 | Symptoms and signs involving the circulatory and respiratory systems |
| GI symptoms | | | | |
|  | **Outcome definition** | | | |
|  | | Diagnosis | UMLS:ICD10CM:R10-R19 | Symptoms and signs involving the digestive system and abdomen |
|  |  |  |  |  |
|  |  |  |  |  |
| Nervous and MSK symptoms | | | | |
|  | **Outcome definition** | | | |
|  | | Diagnosis | UMLS:ICD10CM:R25-R29 | Symptoms and signs involving the nervous and musculoskeletal systems |
|  | |  |  |  |
| Anxiety/mood disorder/behavioral syndrome/cognitive disorders | | | | |
|  | **Outcome definition** | | | |
|  | | Diagnosis | UMLS:ICD10CM:F30-F39 | Mood [affective] disorders |
|  | | Diagnosis | UMLS:ICD10CM:F40-F48 | Anxiety, dissociative, stress-related, somatoform and other nonpsychotic mental disorders |
|  | | Diagnosis | UMLS:ICD10CM:F50-F59 | Behavioral syndromes associated with physiological disturbances and physical factors |
|  | | Diagnosis | UMLS:ICD10CM:R40-R46 | Symptoms and signs involving cognition, perception, emotional state and behavior |

| Diagnostic imaging tests | | | | |
| --- | --- | --- | --- | --- |
|  | **Outcome definition** | | | |
|  | | Procedure | UMLS:CPT:1010252 | Diagnostic Radiology (Diagnostic Imaging) Procedures |
|  |  |  |  |  |
| Diagnostic CV test - echocardiogram and ambulatory rhythm monitor | | | | |
|  | **Outcome definition** | | | |
|  | | Procedure | UMLS:CPT:1013050 | Echocardiography Procedures |
|  | | Procedure | UMLS:CPT:1020409 | Cardiovascular Monitoring Services |
|  |  |  |  |  |
| Anxiety/mood disorder/behavioral syndrome/cognitive disorders | | | | |
|  | **Outcome definition** | | | |
|  | | Diagnosis | UMLS:ICD10CM:F30-F39 | Mood [affective] disorders |
|  | | Diagnosis | UMLS:ICD10CM:F40-F48 | Anxiety, dissociative, stress-related, somatoform and other nonpsychotic mental disorders |
|  | | Diagnosis | UMLS:ICD10CM:F50-F59 | Behavioral syndromes associated with physiological disturbances and physical factors |
|  | | Diagnosis | UMLS:ICD10CM:R40-R46 | Symptoms and signs involving cognition, perception, emotional state and behavior |
|  |  |  |  |  |
| Ambulatory or virtual visit | | | | |
|  | **Outcome definition** | | | |
|  | | Visit | UMLS:HL7V3.0:VisitType:AMB | Visit: Ambulatory |
|  | | Visit | UMLS:HL7V3.0:VisitType:VR | Visit: Virtual |
|  |  |  |  |  |

## Propensity Score Matching

Propensity score matching was performed on 42 characteristic(s).

| **Cohort 1 and cohort 2 patient count before and after propensity score matching** | | | | | |
| --- | --- | --- | --- | --- | --- |
|  | | Cohort | Patient count before matching | | Patient count after matching |
|  | | 1 - CVD, Yes Pax, Dec 31 2022 | 26,594 | | 26,593 |
|  | | 2 - CVD, No Pax, Dec 31 2022 | 218,684 | | 26,593 |
| **Propensity score density function - Before and after matching (cohort 1 - purple, cohort 2 - green)** | | | | | |
|  |  | 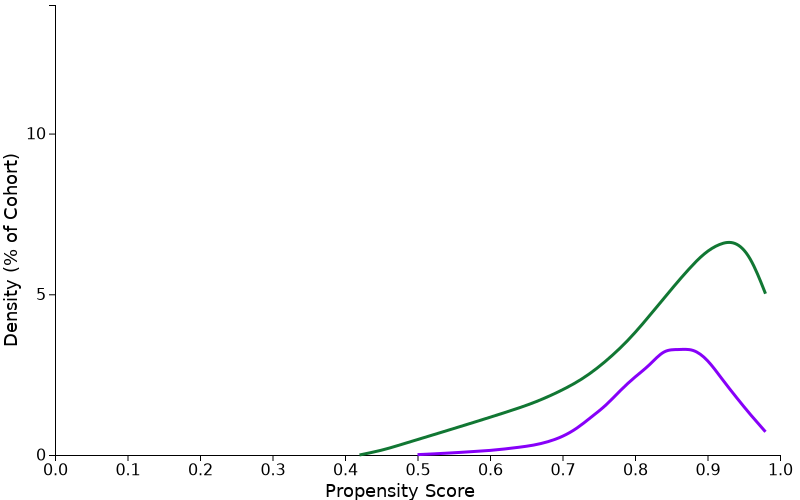 | | 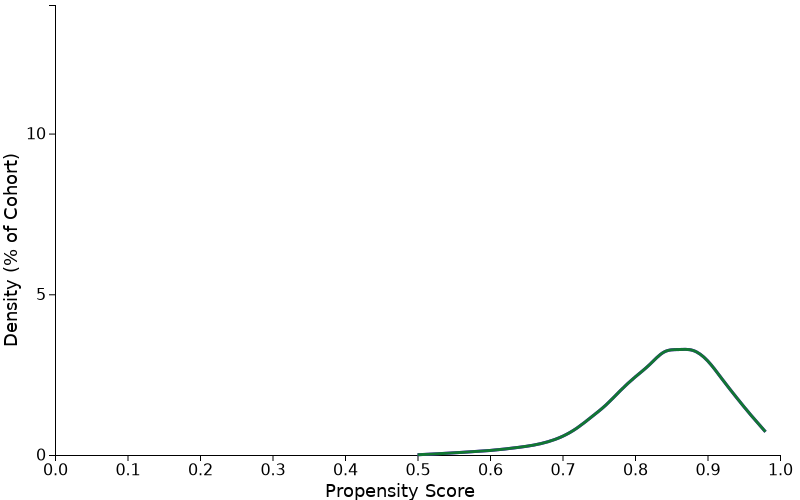 | |
